# Transcriptomic Heterogeneity in Cancer as a Consequence of Dysregulation of the Gene–Gene Interaction Network

**DOI:** 10.1007/s11538-015-0103-7

**Published:** 2015-09-16

**Authors:** Wessel N. van Wieringen, Aad W. van der Vaart

**Affiliations:** Department of Epidemiology and Biostatistics, VU University Medical Center, P. O. Box 7057, 1007 MB Amsterdam, The Netherlands; Department of Mathematics, VU University Amsterdam, De Boelelaan 1081a, 1081 HV Amsterdam, The Netherlands; Department of Mathematics, Leiden University, P. O. Box 9512, 2300 RA Leiden, The Netherlands

**Keywords:** Entropy, Gaussian graphical model, Gene expression, Multivariate normality, Pathway, 62H99, 94A15

## Abstract

Many pathways are dysregulated in cancer. Dysregulation of the regulatory network results in less control of transcript levels in the cell. Hence, dysregulation is reflected in the heterogeneity of the transcriptome: the more dysregulated the pathway, the more the transcriptomic heterogeneity. We identify four scenarios for a transcriptomic heterogeneity increase (i.e., pathway dysregulation) in cancer: (1) activation of a molecular switch, (2) a structural change in a regulator, (3) a temporal change in a regulator, and (4) weakening of gene–gene interactions. These mechanisms are statistically motivated, explored in silico, and their plausibility to occur in vivo illustrated by means of oncogenomics data of breast cancer studies.

**Electronic supplementary material:**

The online version of this article (doi:10.1007/s11538-015-0103-7) contains supplementary material, which is available to authorized users.

## Introduction

Cancer is not one but a collection of many diseases (Insight section of Nature 501, 2013). It is different between and within patients. A severe consequence of this heterogeneity may be found in the many cancers that relapse after treatment [confer the review papers (Hart and Fidler 1981; Dexter and Leith 1986; Heppner and Miller 1983; Stingl and Caldas 2007; Marusyk and Polyak 2010; Shackleton et al. 2009; Pinto et al. 2013)]. In the face of extreme selection pressure due to treatment, some cancer cells may survive, enabling the tumor to recover. Between-cell heterogeneity can thus increase the probability of a cancer being resistant to treatment [mathematically, Goldie and Coldman (1978) and Demetrius et al. (2004) provide theoretical underpinning of this phenomenon]. An ad hoc solution to tackle this heterogeneity currently tested by medical researchers is to combine treatments. Better treatments may arise from a more profound understanding of the mechanisms that contribute to the heterogeneity of cancer, as they offer suggestions about the different ways by which tumors overcome treatment.

Heterogeneity arises during the evolution of cancer (Nowell 1976). A progenitor cell acquires a cancerous mutation which gives the cell a selective advantage within the microenvironment of the tissue. The clones and subclones of the progenitor cell accumulate further genetic abnormalities. This evolutionary process breeds a heterogeneous population of cells that form the tumor. This explanation of cancer heterogeneity is centered around the DNA and ignores contributions from other parts of the cancer cell. Here we investigate, from the perspective of the regulatory network, how the transcriptome may contribute to the heterogeneity of cancer.

The cellular regulatory network consists of a set of connected pathways. A pathway is a chain of chemical reactions occurring in the cell. The common conceptualization of a pathway is that of a collection of genes that interact in order to fulfill a particular cellular function. This conceptualization motivates the treatment of pathways as networks. In these networks, genes are represented by nodes and the interactions between genes by edges. When (for instance) modeling the gene expression levels, denoted $\mathbf{Y}$, of a pathway in equilibrium by a *p*-variate normal distribution, i.e., $\mathbf{Y} \sim {\mathcal {N}}(\varvec{\mu }, {\varvec{\Sigma }})$, the “gene–gene interaction” graph underlying this multivariate Gaussian process is given by the network. Nonzero partial correlations (proportional to elements of ${\varvec{\Sigma }}^{-1}$) between the variates (genes) of $\mathbf{Y}$ coincide with the presence of edges (interactions) in the network.

The expression levels of a pathway’s genes need to be well controlled for the cell to function properly. Normal and cancer cells, however, exhibit many differentially expressed genes. Abnormal expression levels in the cancer cell may dysregulate pathways, by inhibiting or stimulating them (Vogelstein and Kinzler 2004). This dysregulation may affect the cell’s fitness (i.e., its ability to proliferate).

Entropy is a measure of heterogeneity. To appreciate this note that the (differential) entropy of a *p*-variate normal random variable $\mathbf{Y}$ is given by the logarithm of the determinant of its covariance matrix:

$$\begin{aligned} H(\mathbf{Y})= & {} -\int _{-\infty }^{\infty } \cdots \int _{-\infty }^{\infty } f_{\mathbf{Y}}(\mathbf{y}) \, \log [ f_{\mathbf{Y}}(\mathbf{y}) ] \, {\hbox {d}}\mathbf{y} \, \, \, = \, \, \, \log ( | {\varvec{\Sigma }} | ). \end{aligned}$$

The determinant of ${\varvec{\Sigma }}$ is equal to the product of the eigenvalues of ${\varvec{\Sigma }}$. It thus equals the volume of a *p*-dimensional ellipsoid spanned by the eigenvectors of ${\varvec{\Sigma }}$ and with lengths of its edges equal to the eigenvalues. As such, the determinant of the covariance matrix is a measure of the spread of the random variable $\mathbf{Y}$. Hence, there is a one-to-one relation between entropy and heterogeneity (the convex logarithmic transformation does not affect this).

Here we interpret entropy (and thus heterogeneity) as a measure of dysregulation. This is motivated as follows. For a pathway to fulfill its function in the cell, its gene expression levels cannot vary randomly. The transcript levels must be regulated, causing them to stay within certain boundaries. A “healthy” pathway’s gene expression data must therefore be concentrated in a subspace of the space of all possible (in a statistical sense) transcript levels. In contrast, gene expression levels of a dysregulated pathway can be expected to be less orderly and more spread. Dysregulation of a pathway in a cancer cell is likely to be reflected in the heterogeneity of its gene expression levels and will then be larger than that of a healthy cell. Hence, entropy is positively associated with dysregulation. Such an interpretation of entropy is long current in the field of control theory, where the controllability of a system is negatively associated with the entropy (Rajapakse et al. 2011).

From the definition, it is clear that the estimation of entropy may proceed through the calculation of the sample covariance matrix and its determinant. When dealing with high-dimensional data, the sample covariance matrix is replaced by a penalized counterpart, as done previously for the estimation of entropy (Van Wieringen and Van der Vaart 2011). The employed penalized covariance estimate is $\hat{{\varvec{\Sigma }}}(\lambda ) = (1-\lambda ) \mathbf{S} + \lambda \mathbf{T}$ as originally proposed in Ledoit and Wolf (2004) and popularized in Schäfer and Strimmer (2005). In this estimator $\lambda \in [0, 1]$ is the penalty parameter and $\mathbf{T}$ a user-specified target matrix, taken to be diagonal with $\text{ diag }(\mathbf{T}) = \text{ diag }(\mathbf{S})$. The penalized covariance estimate is thus a weighted average of sample covariance matrix and target matrix. When $\lambda$ increases, the penalized covariance estimate is shrunken toward the target matrix $\mathbf{T}$. The penalty parameter is chosen to minimize the sum of the mean squared errors of the elements of $\hat{{\varvec{\Sigma }}}(\lambda )$. The moments involved in the mean squared error are estimated from the data at hand. Naturally, larger sample sizes yield more reliable estimates of these moments (and, consequently, of the choice of the penalty parameter) than smaller ones. In the remainder of this work, we compare the entropy between groups, which may be of different sample size. To minimize the influence of the choice of the penalty parameter on entropy comparisons, equally sized subsamples are used.

In previous work (Van Wieringen and Van der Vaart 2011), we observed that transcriptomic heterogeneity (operationalized as entropy) in cancer tissue systematically exceeds that of corresponding normal tissue. In particular, the cross-sectional oncogenomics studies analyzed in Van Wieringen and Van der Vaart (2011) exhibited a concordant increase with the progression of the disease. Independently and by different means, it was concluded in Schramm et al. (2010) that the “regulatory entropy” of cancer cells exceeds that of normal cells. In Teschendorff and Severini (2010), a higher transcriptomic heterogeneity (entropy) in metastasized cancer over healthy controls was also noted. In follow-up work (West et al. 2012; Banerji et al. 2013; Teschendorff et al. 2014; Banerji et al. 2015), the authors of Teschendorff and Severini (2010) confirmed that “cancer is characterized by an increase in network entropy” and observed the entropy increase in other types of cellular transformation. An abundance of similar and related observations is quoted by the review papers (Berretta and Moscato 2010; Tarabichi et al. 2013). Moreover, it has even been proposed (Berretta and Moscato 2010) to add “entropy increase” to the hallmarks of cancer (Hanahan and Weinberg (2000).

In this paper, we explore from the perspective of the cellular regulatory network, using both mathematical modeling and publicly available experimental data, how a transcriptomic heterogeneity surge may come about. We present several mechanisms that may lead to an increase in the cancer cell’s transcriptomic heterogeneity. The mechanisms are mathematically motivated by analytic results (with proofs given in the Supplementary Material, henceforth SM). In simulation studies, it is assessed how topological features of the regulatory network influence the mechanisms for heterogeneity increase. Finally, oncogenomics data from breast cancer studies are used to illustrate that the discussed mechanisms for transcriptomic heterogeneity increase indeed occur in the cancer cell.

## Switches

**Fig. 1:**
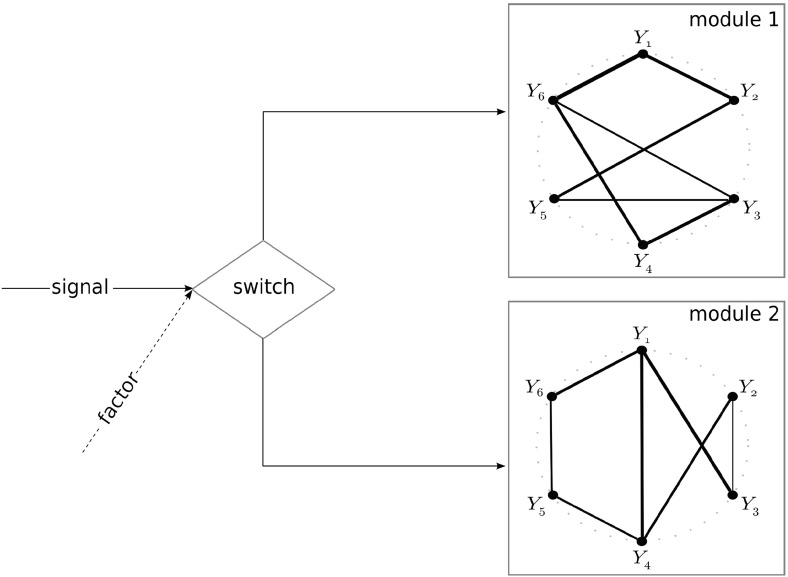
A molecular switch, influenced by a factor, between two regulatory modules

The increase in transcriptomic heterogeneity with the progression of cancer may be explained by the presence of switches in the regulatory network. A switch enables the cell to change between regulatory modules (Fig. 1), leading to differential gene expression patterns. Which module is activated by the switch depends on, e.g., an environmental factor. An example of a switch may be found in the MAPK pathway, where growth conditions determine which MAPK protein is produced (Zalatan et al. 2012). Downstream the proteins may activate different regulatory modules. The modules may result in different gene expression patterns. If some cancer cells switch to a module that yields a different transcriptomic entropy, the heterogeneity of the whole population changes.

Regulatory switches may give rise to the multiple distinct cancer subgroups of one tissue and as such contribute to the increase in the transcriptomic entropy. To demonstrate this, we assume that the cancer samples in a genomic study originate from $K \ge 2$ subgroups. The subgroup information is considered unknown. The gene expression profile of a regulatory module in sample *i*, represented by the *p*-dimensional random variable $\mathbf{Y}_i$ with each element corresponding to a gene in the module, may then be modeled by a finite mixture model:

1 $$\begin{aligned} \mathbf{Y}_i\sim & {} \sum _{k=1}^K \tau _k \, {\mathcal {N}} ( \varvec{\mu }_k, {\varvec{\Sigma }}_k), \end{aligned}$$

with mixing proportions $\tau _k \ge 0$ (which sum to one) and mixture components ${\mathcal {N}} ( \varvec{\mu }_k, {\varvec{\Sigma }}_k)$. Each mixture component describes how the expression data within a subgroup are distributed. Furthermore, we assume that after normalization, we have:

2 $$\begin{aligned} E(\mathbf{Y}_i)= & {} \sum _{k=1}^K \tau _k \varvec{\mu }_k \, \, \, = \, \, \, \mathbf{0}. \end{aligned}$$

This assumption simplifies the argument below, but does not affect its conclusion.

The following proposition (proof in SM A) relates the entropy of the mixed distribution (1) to that of an unmixed distribution.

### **Proposition 1**

Let $\mathbf{Y}$ be a *p*-variate random variable in ${\mathbb {R}}^p$, $f_z(\mathbf{Y}) = f(\mathbf{Y} | z)$ be a density for every *z* in a domain *D*. Then, if *G* is a probability distribution on *D* and $f_G(\mathbf{Y}) = \int _D f(\cdot | z) \, dG(z)$:

$$\begin{aligned}& - \int _{{\mathbb {R}}^p} f_G(\mathbf{Y}) \log [ f_G(\mathbf{Y}) ] \, \mathrm{d} \mathbf{Y} \ge -\int _D \int _{{\mathbb {R}}^p} f_z(\mathbf{Y}) \log [ f_z(\mathbf{Y}) ] \, \mathrm{d} \mathbf{Y} \, \mathrm{d} G(z). \end{aligned}$$

The proposition is formulated in terms of an unspecified mixing distribution *G*, which may be chosen to be discrete as in mixture distribution (1).

Proposition 1 teaches us that transcription levels of a heterogeneous population (as comprised by the individuals with cancer of a given type of tissue) are less concentrated than those of a homogeneous population (formed by the individuals with healthy tissue). The heterogeneity may be due to activation of a different regulatory modules by switches. Proposition 1 relates two features of these regulatory modules to transcriptomic heterogeneity. To see this, apply Proposition 1 to *p*-variate random variables $\mathbf{Y}$ following the mixture distribution (1) with mean zero, assumption (2) and $\mathbf{X} \sim {\mathcal {N}}( \mathbf{0}, {\varvec{\Sigma }})$. Then, $H(\mathbf{Y}) \ge H(\mathbf{X})$, if either:

1. there are $k_1, k_2 \in \{ 1, \ldots , K \}$ such that $\varvec{\mu }_{k_1} \not = \varvec{\mu }_{k_2}$ and ${\varvec{\Sigma }}_k ={\varvec{\Sigma }}$ for all *k*, or
2. $$|{\varvec{\Sigma }}_k | \ge |{\varvec{\Sigma }}| $$ for every $$k \in \{ 1, \ldots , K \}$$.

The first scenario (1) boils down to differential expression (of one or more genes) between any two regulatory modules controlled by the switch. The second scenario (2) requires an heterogeneity increase in one of the regulatory modules (for which later sections provide clues).

It needs empirical investigation whether the two scenarios that may cause the heterogeneity difference (Proposition 1) indeed occur in cancer. With respect to the first scenario, it is beyond doubt that subpopulations (possibly induced by the switch) exhibit differential expression. Any specialization of the cancer cell will be hard to imagine without changing expression levels during the course of the disease. Hence, we do not illustrate the first scenario with data. Instead, we point out a connection between differentially expressed genes and their role in the pathway. Wachi et al. (2005) observe that differentially expressed genes in lung cancer are more likely to have a large number of edges in the regulatory network. Independently, Jonsson and Bates (2006) point out that cancer genes tend to be more highly connected. Although these claims need further substantiation from independent studies, they hint at a more prominent role of central genes in the increase in transcriptomic heterogeneity.

We turn to the second delineated scenario for an heterogeneity surge as implicated by Proposition 1: an heterogeneity increase in a subgroup (due to switching to a different regulatory module). To our knowledge, no publicly available data from an oncogenomics study are at hand to evaluate this properly. As a surrogate, we investigate whether the heterogeneity differs between subgroups (a consequence of the cause). This is done in five breast cancer studies (available via the Bioconductor repository, SM B) that have been uniformly preprocessed with widely accepted methodology [confer (Schröder et al. 2011) for details]. Each study profiled the transcriptome of the samples included. In addition, information on the estrogen receptor (ER) status is available. The ER status can be positive (or negative) referring to the involvement (or not) of estrogen receptors, a group of proteins that may regulate the activity of many genes (Björnström and Sjöberg 2005), in the breast tumor. Prognosis is poorer for ER-negative tumors, which is (partially) due to the availability of reasonably successful hormone treatment for the ER-positive tumors. Many pathways cross-talk with ER status, in particular the Notch and TGF$\beta$ pathways (Band and Laiho 2011). This cross talk leads to differential expression patterns between ER-positive and ER-negative tumors.

A switch activating estrogen receptors seems a plausible underlying mechanism causing these differences in expression. Hence, comparison of the transcriptomic heterogeneity of the ER-positive and ER-negative groups may indicate whether the second cause (as delineated in the interpretation of and directly following Proposition 1) is a biologically plausible scenario.

The estrogen receptor refers to a group of proteins that, when active, regulate the activity of many genes (Björnström and Sjöberg 2005). This group of proteins thus forms a molecular switch.

**Fig. 2:**
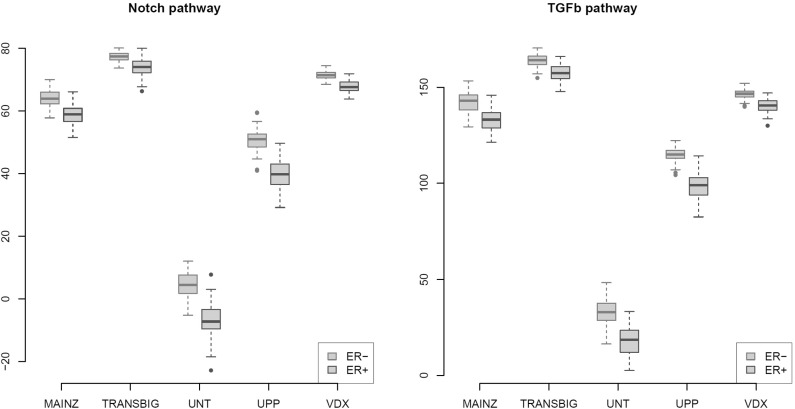
*Boxplot* of entropy estimates versus ER status. *Left panel* Notch pathway; *right panel* TGF$\beta$ pathway (Color figure online)

We compare the transcriptomic entropy of the ER-positive and ER-negative groups in both the Notch and TGF$\beta$ pathways using all breast cancer data sets, which have been limited to the genes present on the corresponding microarray platform and that map to these pathways as defined by the curated KEGG repository (Ogata et al. 1999) using their Entrez IDs. To ensure that the difference in samples size between the ER groups does not affect the comparison (a possibility pointed out in the introductory section), the groups are subsampled with equal sample size, which is set at 90 % of the smallest group. We subsample 500 times to average out the random variation due to the sampling. At each draw, the samples from the ER groups form a subsampled version of the original data set. From the subsampled data set, the shrinkage parameter for shrunken covariance matrix estimation is estimated (Schäfer and Strimmer 2005). This estimated shrinkage parameter is then used to estimate the transcriptomic entropy of the individual groups (Van Wieringen and Van der Vaart 2011). Figure 2 shows the resulting 500 entropy estimates for the ER groups in the data sets for the Notch and TGF$\beta$ pathways. Conditional on the full data set, these estimates indicate the distribution of entropy in each ER group. In both pathways, the transcriptomic entropy is (in each of the five data sets) lower in the ER-positive group. Hence, the ER-negative group exhibits more heterogeneity than the ER-positive group. This is in line with the observation that more cancer heterogeneity is associated with a poorer outcome. In summary, there may be an alternative (to differential expression) route to increase transcriptomic heterogeneity, namely via a heterogeneity surge in a subpopulation delineated by the activation of a different regulatory module. The next sections provide clues how the larger transcriptomic heterogeneity of this module may arise.

## Increased Variation in an Expression Regulator

Variation in gene expression levels may surge with increased fluctuations in factors like DNA copy number that influence transcription. Consequently, if during the progression of the disease a cancer cell switches from a regulatory module without to one with genomic aberrations, the transcriptomic heterogeneity may increase. Indeed, as we have shown previously in Van Wieringen and Van der Vaart (2011), an increase in genomic heterogeneity is propagated to the transcriptomic level. A plausible model for the interaction of these two molecular levels is provided (among others) in Van Wieringen and Van de Wiel (2014). The model is briefly recapitulated here to explain concordant genomic and transcriptomic heterogeneity increase. In ongoing work, we extend that model to describe the microRNA–mRNA interactions. In the mathematical argumentation for the transcriptomic heterogeneity increase due to genomic aberrations as provided below the role of latter may be replaced by that of microRNAs.

**Fig. 3:**
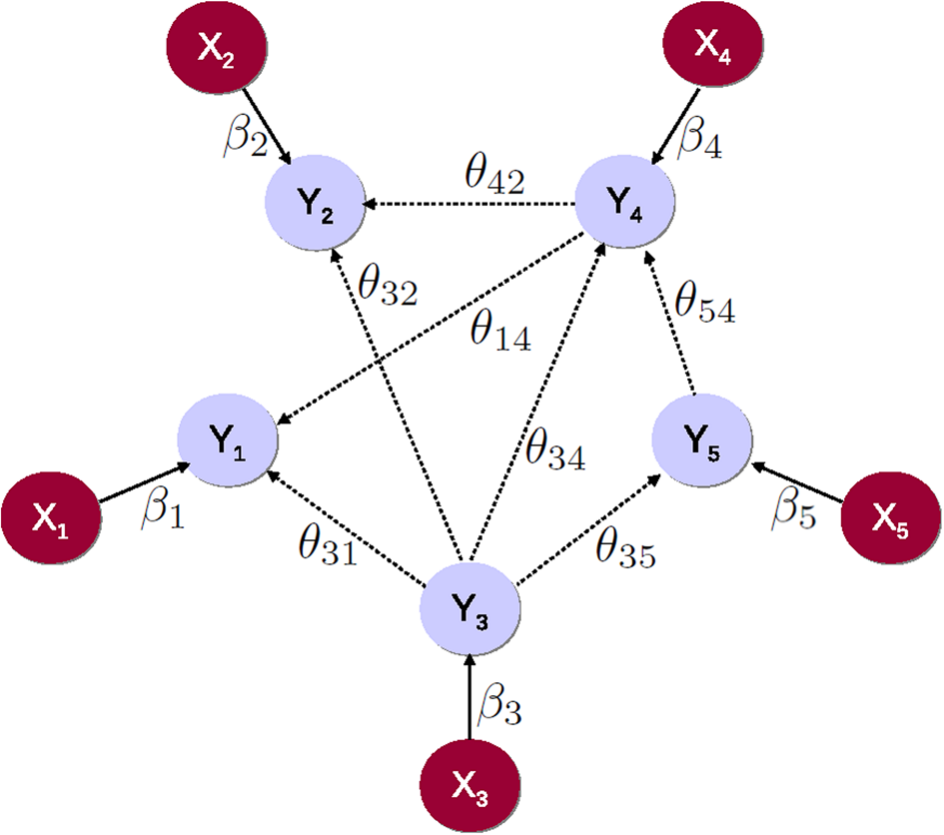
Schematic representation of Model (3), describing the interplay between DNA copy number aberrations and gene expression within a regulatory network. The *solid arrows* correspond to the *cis*-effect ($\varvec{\beta }$) of the gene dosage, whereas the elements of ${\varvec{\Theta }}$ are displayed as *dashed arrows* (Color figure online)

Let $\mathbf{X}$ and $\mathbf{Y}$ be *p*-dimensional vectors of DNA copy number and gene expression information, respectively. The relation between the two may be described by the rate equation:

$$\begin{aligned} \nabla _t \mathbf{Y}= & {} \mathbf{f}(\mathbf{Y}) - \varvec{\gamma }\circ \mathbf{Y} + \varvec{\beta }\circ \mathbf{X}, \end{aligned}$$

where the $\circ$-operator denotes the Hadamard product, $\varvec{\gamma }$ the decay rate of the mRNAs, and $\varvec{\beta }$ the effect of DNA copy number changes on the expression levels. This equation links the change in gene expression with time to the *p*-dimensional vector-valued transcription function $\mathbf{f}(\cdot )$, the decay rate (the second summand on the right-hand side), and the *cis*-effect of the genomic aberration (third summand). In order for the rate equation to be applicable to data from integrative genomic studies, where the two molecular levels of a random sample are measured in an observational experimental setup, two simplifying assumptions are made: (1) a steady state and (2) a linear form of $\mathbf{f}(\cdot )$ (although not strictly necessary). After regrouping of terms and the introduction of an error term $\varvec{\varepsilon }$, with $\varvec{\varepsilon }\sim {\mathcal {N}}(\mathbf{0}, {\varvec{\Sigma }})$, we arrive at:

3 $$\begin{aligned} {\varvec{\Theta }} \mathbf{Y}= & {} \varvec{\beta }\circ \mathbf{X} + \varvec{\varepsilon }, \end{aligned}$$

where ${\varvec{\Theta }}$ contains the edges between the genes in the regulatory network. For example, an element $({\varvec{\Theta }})_{j_1, j_2}$ represents the effect of gene $j_1$ on gene $j_2$. Model (3) is visually portrayed in Fig. 3. For the identifiability and estimation of Model (3), refer to Van Wieringen and Van de Wiel (2014).

To see how Model (3) may explain that an increase in genomic heterogeneity could lead to an increase in the heterogeneity of gene expression levels, rewrite the model to: $\mathbf{Y} \, | \, \mathbf{X} \sim {\mathcal {N}} \left( {\varvec{\Theta }}^{-1} [ \varvec{\beta }\circ \mathbf{X}], {\varvec{\Theta }}^{-1} {\varvec{\Sigma }} [{\varvec{\Theta }}^{-1}]^T\right)$. The unconditional variance of $\mathbf{Y}$ is then given by:

$$\begin{aligned} \text{ Var }(\mathbf{Y}) = \left[ {\varvec{\Theta }}^{-1} \circ (\mathbf{1}_p \otimes \varvec{\beta }^T)\right] \, \text{ Var }(\mathbf{X}) \, \left[ {\varvec{\Theta }}^{-1} \circ (\mathbf{1}_p \otimes \varvec{\beta }^T)\right] ^T + {\varvec{\Theta }}^{-1} {\varvec{\Sigma }} \left[ {\varvec{\Theta }}^{-1}\right] ^T. \end{aligned}$$

First and second summands on the right-hand side are (semi)-positive and positive definite, respectively. Corollary 18.1.7 of Harville (2008) then ensures that $\text{ det }[\text{ Var }(\mathbf{Y})] \ge \text{ det }\left( {\varvec{\Theta }}^{-1} {\varvec{\Sigma }} [{\varvec{\Theta }}^{-1}]^T \right)$, with equality holding only if the covariance of $\varvec{\beta }\circ \mathbf{X}$ is zero. In particular, the transcriptomic entropy is unaffected by DNA copy number aberrations if the variation in $\mathbf{X}$ is nil (no aberrations), or if DNA copy number aberrations do not affect gene expression levels.

If genomic aberrations occur and affect the pathway’s transcription levels (i.e., $\varvec{\beta }\circ \mathbf{X} \not = \mathbf{0}_{p \times 1}$), does it matter which gene is aberrated? Indeed, as genomic aberrations are inherited by daughter cells and their prevalences vary over genes, different prevalences (roughly, evolutionary selection frequencies) of DNA copy number aberrations suggest different fitness contributions. Should genomic aberrations have an effect on the cancer cell, their prevalence differences have to manifest themselves at the transcriptomic level of the pathway. To investigate this, we study in silico the relation between the effect of a gene’s genomic entropy and its node degree. The motivation behind the choice of this topological feature stems from the observation that an oncogene may be a transcription factor (Look 1997). Transcription factors often contribute to the regulation of many other genes and tend to be highly connected. For instance, the oncogene MYC is a transcription factor. MYC is known to be often amplified in many cancers (Futreal et al. 2004). This often leads to the deregulation of the cell cycle, among others stimulating cellular proliferation (Dang et al. 1999). In the simulation (more details in SM C), the genomic heterogeneity (present in $\mathbf{X}$) of the genes—one at a time—is increased and its effect on the transcriptomic entropy of $\mathbf{Y}$ studied by means of Model (3). Relating the node degree of the gene with increased genomic heterogeneity to the resulting increase in transcriptomic entropy then indicates whether a gene’s connectivity modulates the latter. The simulation starts by setting the network size (i.e., the number of nodes) $p=50, 100$ or 250, $\varvec{\beta }= \mathbf{1}_{p \times 1}$ and ${\varvec{\Sigma }} = 0.3 \, \mathbf{I}_{p \times p}$, and sampling a hypothetical regulatory network topology (either small world or scale free). For each node in the sampled hypothetical networks, its degree $d_j$ is determined. Then, the genomic entropy of node *j*, i.e., $[\text{ Var }(\mathbf{X})]_{jj}$, is increased (from zero to one, keeping that of the other nodes fixed at zero). The resulting entropy of $\mathbf{Y}$, denoted $H_j$, is calculated. The $H_j$ are plotted against the node degree $d_j$ (see Fig. 1 of the SM C). The plots indicate an increase in transcriptomic entropy, an increase, however, independent of the affected node’s connectivity. The latter may be counter-intuitive, but can be explained analytically (see SM C). It can also be understood when realizing that Model (3) is a description of the relation between DNA copy numbers and gene expression levels within a pathway in a *closed system at equilibrium*. The system itself is kept constant in the simulation, only the genomic variance of a single gene is increased. The inserted additional variation introduced into the system cannot escape, due to it being in equilibrium and having no sink. Hence, the inserted variation must stay within the system, irrespective of where it had been inserted.

In the previous subsection (Switch), it has been observed that ER${-}$ breast tumors exhibited more heterogeneity than their ER+ counterparts. Model (3) suggests that this may be due to the DNA copy number aberrations. Or, more specifically, due to an increase in the covariance of $\varvec{\beta }\circ \mathbf{X}$. Whether this increase occurs is investigated in three breast cancer data sets (details, including that of all the preprocessing steps that use well-accepted methodology, can be found in SM B). The data sets comprise genomic and transcriptomic profiles and the ER status of all its samples. As in the previous subsection, we restrict ourselves to the Notch and TGF$\beta$ pathway. To this end, the three data sets have been limited to the genes present on the corresponding microarray platforms and that map to these pathways as defined by the curated KEGG repository (Ogata et al. 1999) using their Entrez IDs.

**Fig. 4:**
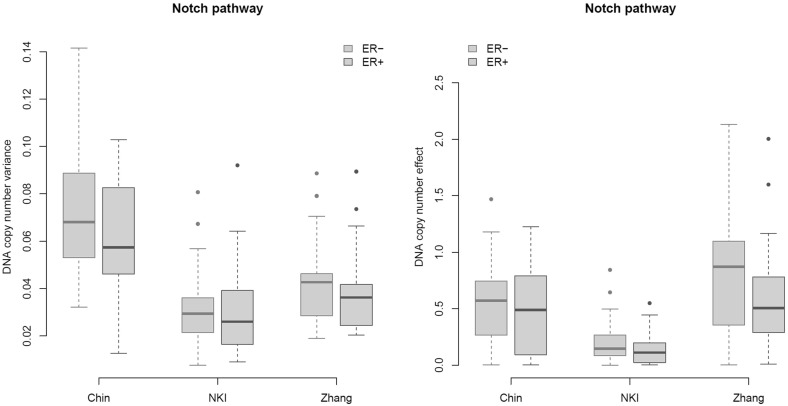
*Left panel* *boxplots* (per data set and ER status) of the variances of DNA copy number of the genes comprising the Notch pathway. *Right panel* *boxplots* (per data set and ER status) of the estimated DNA copy number effect on gene expression (parameters $\beta _j$ of Model 3) of the genes comprising the Notch pathway (Color figure online)

We first concentrate on the variance of the DNA copy number, which is compared between the ER groups in two ways. The first approach assesses whether there is a genomic entropy difference. This is done exactly as in the previous subsection (Switch) for the transcriptomic entropy, but now the gene expression data are replaced by DNA copy number data. This shows a genomic entropy difference between the ER groups concordant with that observed in the transcriptome (plots not shown). Secondly, the variance in DNA copy number between ER${-}$ and ER+ is compared gene-wise. For the Notch pathway (confer SM E for the TGF$\beta$ pathway), these estimated variances are compared between the two ER statuses by means of boxplots (Fig. 4, left panel). Hence, both the univariate and multivariate perspectives reveal a genomic variance in the ER-negative group that is (somewhat) greater than or comparable to that in the ER positives.

The increase in the covariance of $\varvec{\beta }\circ \mathbf{X}$ may also be due to a difference in the gene dosage effect $\varvec{\beta }$ between the groups, which is now investigated. Hereto Model (3) is fitted for both ER groups to their DNA copy number and gene expression data. The model is fitted using the method of Van Wieringen and Van de Wiel (2014) which uses an equation-by-equation $L_1$-penalized least squares approach (only penalizing ${\varvec{\Theta }}$ and leaving $\varvec{\beta }$ unpenalized). For a given penalty $\lambda _1$, fitting Model (3) yields among others a topology (the selected incoming and outgoing edges as reflected in the nonzero elements of ${\varvec{\Theta }}$) and biased estimates of the *cis*-effect $\varvec{\beta }$. To obtain “unbiased” estimates of the *cis*-effect $\varvec{\beta }$, the model is refitted incorporating the found topology. Hereto the same method of Van Wieringen and Van de Wiel (2014) is used with $\lambda _1 = 0$ or $\lambda _1 = \infty$ for each selected or nonselected edge, respectively. The latter ($\lambda _1 = \infty$) results in estimates equal to zero, while the former ($\lambda _1 = 0$) does not constrain the parameter estimate at all. We repeat the above for a grid of $\lambda _1$’s. The grid is constrained to those $\lambda _1$ that result in a sparse network (i.e., having between 1 and 10 % of the total number of possible network edges). The thus estimated $\beta$’s (with a specific $\lambda _1$ from the aforementioned domain) are compared between the groups by means of boxplots (Fig. 4, right panel, Notch pathway only; see SM E for the results of the TGF$\beta$ pathway). Over the data sets and in both pathways, the ER- group shows slightly larger DNA copy number effects ($\varvec{\beta }$). This conclusion is unaffected by the choice of the penalty parameter as the estimated $\varvec{\beta }$ changes little over the grid of $\lambda _1$’s, due to the fact that (a) $\varvec{\beta }$ itself is not penalized and (b) the preferred sparse models include only few covariates in each regression equation of Model (3) with shrunken (i.e., small) estimates of their regression coefficients.

In summary, more heterogeneity in a regulator (like DNA copy number) may, if Model (3) is a reasonable approximation, lead to more heterogeneity downstream in the transcriptome. The breast cancer example above suggests that (part of) the entropy difference between the ER+ and ER${-}$ groups may be attributed to DNA copy number. Changes in the regulator need not be the only source responsible for the entropy difference. In particular, when using the fitted Model (3) to correct for DNA copy number and obtain the “residual” gene expression, the ER- group still exhibits a larger entropy than the ER+ group. This suggests that there may be additional mechanisms contributing to heterogeneity.

## Disturbances

**Fig. 5:**
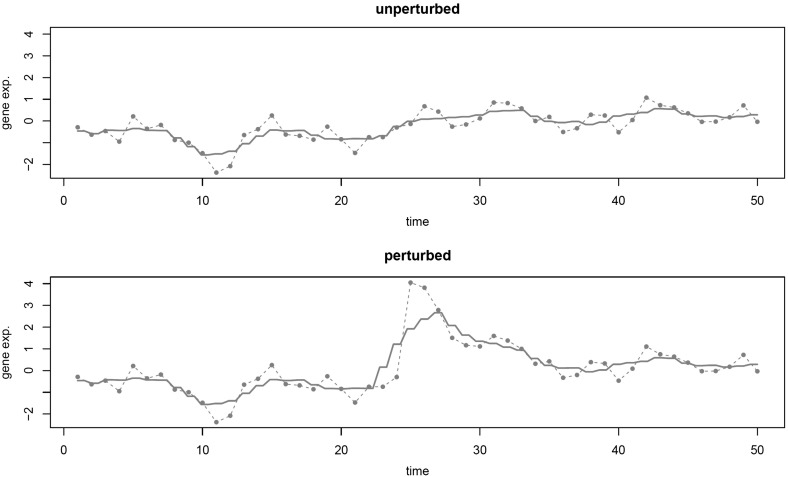
Illustration of the effect of a disturbance. Artificial time series data of the expression levels of a three-gene pathway are generated in accordance with Model (4) (full parametric details given in SM F). The (unperturbed) data, representing gene expression levels of the first gene of the pathway, are shown in the *top panel*. The *lower panel* contains the perturbed data of this gene, generated in accordance with Model (5) with the same innovations as the unperturbed data except for the disturbance. The disturbance occurs at time point $t=25$. The *dashed line* connects the observation. The *solid line* is a moving average smoothing of the data (Color figure online)

The previous section attributes the surge in transcriptomic heterogeneity to switching to a regulatory module with changes in its DNA copy number. If such changes may cause this heterogeneity surge, one expects temporary changes to have a similar effect. Indeed, disturbances of the cellular regulatory network may also cause the transcriptomic entropy increase. This can be witnessed in perturbation experiments, in which the consequences (e.g., at the transcriptomic level) of an internal or external alteration to the cellular regulatory network are studied. An artificial illustration of this is given in Fig. 5. The figure portrays the expression levels of a gene over time, in the situation without and with a disturbance. It is obvious that the disturbed sequence exhibits more variation. A well-known example of such a disturbance is radiation. Exposure to radiation, even at low dose, may cause thyroid cancer (e.g., Ron et al. 1995). Below we provide a statistical underpinning of the effect of a disturbance on the increase in transcriptomic heterogeneity.

To provide a statistical motivation for the heterogeneity increase caused by disturbances, consider an oncogenomics study with a time-course setup. In such a study, a sample (cell line) is followed over time for a certain period and, at multiple time points during this period, is interrogated molecularly. The resulting expression profile at time point *t* is denoted by $\mathbf{Y}_t$. Assume $\mathbf{Y}_t$ can be modeled by a VAR(1) (first-order vector autoregressive) process:

4 $$\begin{aligned} \mathbf{Y}_{t}= & {} \varvec{\nu }+ \mathbf{A} \mathbf{Y}_{t-1} + \varvec{\varepsilon }_{t}, \end{aligned}$$

where $\varvec{\nu }$ the $p \times 1$ intercept vector, $\mathbf{A}$ a $p \times p$ coefficient matrix, and $\varvec{\varepsilon }_{t}$ a $p \times 1$ vector with the errors. It is assumed that $\varvec{\varepsilon }_{t} \sim {\mathcal {N}}(\mathbf{0}_{p \times 1}, {\varvec{\Sigma }}_{\varepsilon })$, $\text{ Cov }(\varvec{\varepsilon }_{t_1}, \varvec{\varepsilon }_{t_2}) = \mathbf{0}$ if $t_1 \not =t_2$, and $\mathbf{Y}_0 = \mathbf{0}_{p \times 1}$. Introduction of a disturbance at time point $\tau$ modifies Model (4) to:

5 $$\begin{aligned} \mathbf{Y}_{t}^{(\tau )}= & {} \varvec{\nu }+ \mathbf{A} \mathbf{Y}_{t-1} + \varvec{\varepsilon }_{t} + \varvec{\delta }_{\tau } I_{ \{t=\tau \}}, \end{aligned}$$

where $\varvec{\delta }_{\tau } \sim {\mathcal {N}}(\mathbf{0}_{p \times 1}, {\varvec{\Sigma }}_{\delta })$ a $p \times 1$ vector with the disturbances.

We can now formulate the following proposition (with proof in SM G):

### **Proposition 2**

Let $\mathbf{Y}_t$ and $\mathbf{Y}_{t}^{(\tau )}$ be *p*-variate random variables distributed in accordance with Models (4) and (5). Then, $H[\mathbf{Y}_{t}^{(\tau )}] > H(\mathbf{Y}_t)$ and $H[\mathbf{Y}_{t}^{(\tau _1)}] > H[\mathbf{Y}_{t}^{(\tau _2)}]$ if $\tau _2 > \tau _1$.

The proposition tells us that (a) the heterogeneity (which is one-to-one related to entropy) of the undisturbed expression data is smaller (in a positive-definite sense) to that of its disturbed counterpart and (b) that a disturbance that occurs more upstream (in time) leads to larger heterogeneity than a more recent disturbance.

## Weakened Conditional Dependencies

**Fig. 6:**
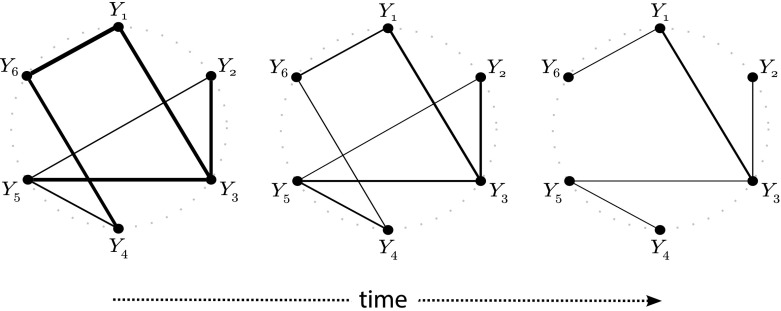
Network changes over time. The fully connected graph on the *left* is a caricature of a regulatory network. The width of the edges is proportional to their hypothesized strengths. Over time, as the disease progresses, interactions between nodes weaken, which is reflected by the decreased width of some of the edges. Eventually, some of these interactions get lost (symbolized by edges that have disappeared), and the graph may even become disconnected

Changes in the architecture of a regulatory network may also affect the cell’s entropy. In particular, as we show here, the weakening of an edge may lead to a surge in the network’s entropy. An extreme case of this phenomenon is the removal of an edge, which indeed may further increase the entropy. Figure 6 illustrates the three cases: the original network (representing the normal, healthy state), the same network with some edges weakened (an early disease state), and, finally, a disconnected network (the late disease state).

We now prove (a similar result does not hold for marginal dependencies, see SM I) that, under normality, decreasing the conditional dependencies in the network between (groups of) nodes conditional of other (groups of) nodes increases the entropy of its associated multivariate distribution. To see this, we study the concentration matrix.

### **Proposition 3**

Let $\mathbf{X} \sim {\mathcal {N}}(\mathbf{0}, {\varvec{\Sigma }}_X)$ and $\mathbf{Y} \sim {\mathcal {N}}(\mathbf{0}, {\varvec{\Sigma }}_Y)$ with equal marginal variances $\text{ diag }({\varvec{\Sigma }}_X) = \text{ diag }({\varvec{\Sigma }}_Y)$. Further, assume that the $p\times p$ partial correlation matrices (i.e., concentration matrices standardized to have a unit diagonal) associated with $\mathbf{X}$ and $\mathbf{Y}$, denoted ${\varvec{\Omega }}_{\varvec{\gamma }^{(x)}}$ and $\mathbf{\Omega }_{\varvec{\gamma }^{(y)}}$ can be both partitioned as $r \times r$ block matrices:

$$\begin{aligned} {\varvec{\Omega }}_{\varvec{\gamma }}= & {} \left( \begin{array}{ccccc} {\varvec{\Omega }}_{11} &{} \gamma _{12} {\varvec{\Omega }}_{12} &{} \ldots &{} \gamma _{1r} {\varvec{\Omega }}_{1r}\\ \gamma _{12} {\varvec{\Omega }}_{12}^T &{} {\varvec{\Omega }}_{22} &{} &{} \vdots \\ \vdots &{} &{} \ddots &{} \vdots \\ \gamma _{1r} {\varvec{\Omega }}_{1r}^T &{} \ldots &{} \ldots &{} {\varvec{\Omega }}_{rr} \end{array} \right) , \end{aligned}$$

with $\varvec{\gamma }=(\gamma _{12}, \ldots , \gamma _{1r}, \gamma _{23}, \ldots , \gamma _{2r}, \ldots , \gamma _{r-1,r}) \in [0,1]^{\frac{1}{2}r (r-1)}$. Then, $\varvec{\gamma }^{(x)} \le \varvec{\gamma }^{(y)}$ (element-wise) implies $H(\mathbf{Y}) \le H(\mathbf{X})$.

Proposition 3 may be interpreted as follows. In a fully connected graph (no element of $\varvec{\gamma }$ is equal to zero), each node is regulated by all other nodes. Hence, they have limited freedom to vary as they please. In a fully disconnected network (all elements of $\varvec{\gamma }$ are equal to zero), each node behaves independently, unconstrained—in any manner—by any of the other nodes. A similar observation is made by Kauffman (1993) when studying random binary networks. Thus, nodes exhibit less variance (entropy) in a fully connected graph than in an unconnected one. In fact, Proposition 3 shows that a decrease in partial correlation already has the same effect: the weaker the conditional dependency between the nodes, the more room to manoeuver freely, the larger the entropy.

Further insight into Proposition 3 is provided by linking the entropy increase to the nodes (rather than the edges) of the network. Hereto, we consider the situation of a gene knockout. Tumor-suppressor genes, recessive in nature, are often knocked out in cancer (Weinberg 2006). A knockout is equivalent to the removal of all edges of a gene (but this is not fully equivalent as the variance of the expression levels of a knocked out gene will vanish, which need not be the case when only its dependencies are removed). An alternative scenario could be a mutation that inhibits the interaction of the gene with others. The mutation does not prohibit the transcription of the gene. Proposition 3 implies that these scenarios lead to an increase in entropy.

**Fig. 7:**
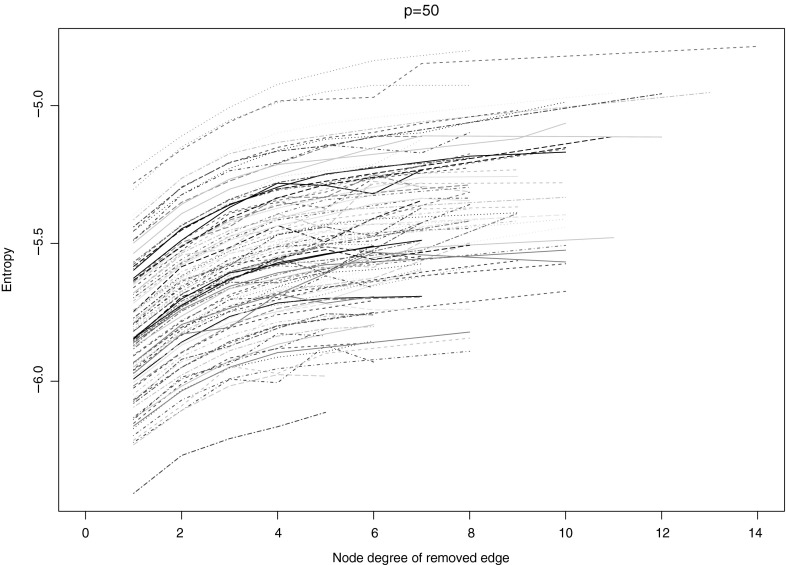
The cubic spline smoothed relationship between the node degree of the node with eliminated conditional dependencies versus the entropy of the resulting $50 \times 50$ dimensional covariance matrix with an underlying scale-free topology. Each *spline* represents the results for an independently drawn covariance matrix. In total, a hundred *splines* are displayed (Color figure online)

We now ask ourselves, from an entropy perspective, whether it matters which gene is silenced. In particular, we investigate—by simulation—the relation between entropy and a gene’s connectivity. This is motivated by the observation of Jonsson and Bates (2006) that cancer genes tend to be more highly connected in the regulatory network. TP53, a well-known tumor-suppressor gene and lost (i.e., silenced) in many cancers, is indeed highly connected (Vogelstein et al. 2000). In the simulation study, the effect of connectivity on the entropy is assessed by eliminating dependencies. Starting point of the simulation is a graph (either small world or scale free) and an associated covariance matrix ${\varvec{\Sigma }}$. For node *j*, we calculate its degree $d_j$, eliminate its edges (conditional dependencies) with the other nodes, and calculate $\log \left( \left| \tilde{{\varvec{\Sigma }}}^{(j)}\right| \right)$ (the entropy), where $\tilde{{\varvec{\Sigma }}}^{(j)}$ is obtained from ${\varvec{\Sigma }}$ by setting all conditional dependencies of node *j* to zero. This is done for each node. Finally, $d_j$ is plotted against $\log \left( \left| \tilde{{\varvec{\Sigma }}}^{(j)}\right| \right)$, where the range of $d_j$ is restricted to the degrees present in the network. Figure 7 shows the results of conditional dependency removal for pathways of $p=50$ genes with a scale-free regulatory network. It reveals a clear monotonously increasing trend: the higher the edge degree of a node, the larger the entropy increase as its conditional dependencies are removed. This holds also for pathways with a small-world topology (refer to the SM J). The plots even suggest a dependence of this relation on the size of the network, but this needs further exploration. In all, the simulation suggests a cancer cell gains most by silencing a highly connected gene, as a it explores different paths of random variation in its evolution and naturally selects the path that leads to a faster entropy increase (Kaila and Annila 2008).

**Fig. 8:**
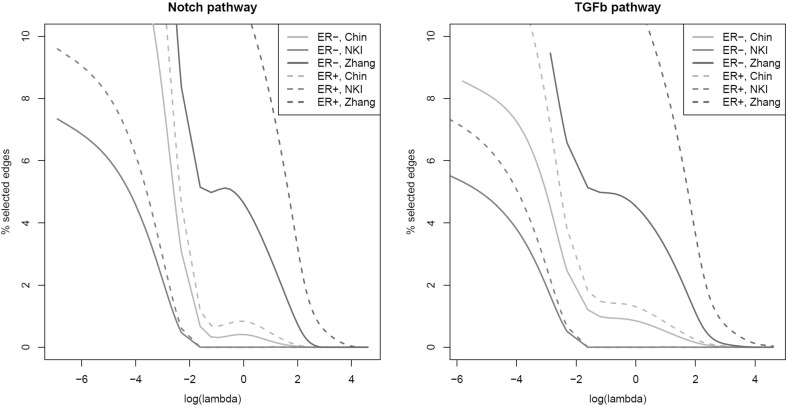
Number of edges present versus penalty parameter, for ER${-}$ (*solid lines*) and ER+ (*dashed lines*) groups for three breast cancer studies (distinguished by *color*). *Left panel* Notch pathway; *right panel* TGF$\beta$ pathway (Color figure online)

It is left to assert whether gene–gene interactions may indeed weaken or vanish in cancer. Hereto we revisit the Notch and TGF$\beta$ pathway data of the previous sections. In Sect. 2, these data revealed a higher transcriptomic entropy in the ER${-}$ group than in the ER+ group. Proposition 3 suggests that the entropy difference may be due to the weakening of gene–gene interactions. To investigate this, we compare between the two estrogen groups their number of conditional dependencies among the genes comprising the pathway. This is done in both the “transcriptome only” (as introduced in Sect. 2) and the integrative oncogenomics data (as introduced in Sect. 3). In the former setting, a standard Gaussian graphical model (as implied by the multivariate normal) describing the relations between the genes is fitted. From the thus fitted model, the nonzero partial correlations (reflecting the relations between the genes) are determined. For the integrative oncogenomics studies, comprising both DNA copy number and gene expression data, Model (3) relating the two molecular levels is fitted. The nonzero elements of the estimate of the matrix ${\varvec{\Theta }}$ with gene-to-gene effects are then studied. For each pathway data set of the previous sections, we subsample repetitively (500 times) an equal number of samples from each estrogen group. This number of samples is set at 90 % of the sample size of the group with the smallest prevalence in the data set. For the “transcriptome only” data of Sect. 2, the number of edges (number of nonzero partial correlations) in each estrogen group is determined for a given penalty parameter $\lambda _1$ using the method of Peng et al. (2009). Similarly, for the integrative oncogenomics studies, comprising both genomic and transcriptomic data, the number of edges (nonzero elements of ${\varvec{\Theta }}$) in both groups is determined for a given penalty parameter $\lambda _1$ using the method of Van Wieringen and Van de Wiel (2014) which fits a sparse version of Model (3). The number of edges found is averaged over the 500 subsamples. The above (for both the “transcriptome only” and the integrative oncogenomics data) is repeated for a grid of $\lambda _1$. In both cases, the penalty parameter grid is chosen such that the resulting number of edges (i.e., the number of nonzero partial correlations, or the number of nonzero off-diagonal elements in $\widehat{{\varvec{\Theta }}}$) is between 1 and 10 % of the total number of possible edges. This range intends to capture only sparse networks, which are believed to be representative of realistic gene–gene interaction networks. The averaged number of selected edges is plotted against the penalty parameter $\lambda _1$ in Fig. 8 for the integrative oncogenomics studies, whereas the plots for the “transcriptome only” data sets are in SM K. The latter suggests that there is no weakening of the gene–gene interaction pattern from one estrogen group to the other in either pathway. However, when taking into account DNA copy number aberrations, it becomes apparent that in both pathways, the number of selected edges in the ER${-}$ samples is lower (over the selected range of $\lambda _1$) than in the ER+ samples (confer the right panel of Fig. 8). This suggests a weaker gene–gene interaction pattern in the ER${-}$ group, which may in turn contribute to the higher transcriptomic entropy/heterogeneity. Hence, it suggests that delineated mechanisms of transcriptomic entropy increase need not always act alone. For instance, in the illustration above only after correction for the effect of genomic abberations did the mechanism of weakened gene–gene interactions become apparent. Two (or more) transcriptomic entropy increasing mechanisms may thus be active simultaneously.

## Conclusion

Transcriptomic heterogeneity increases as cancer progresses. Here we presented several statistically motivated and biologically plausible mechanisms that may explain this surge:

- Activation of molecular switches. Two tangible manifestations of heterogeneity are (a) differential expression between the regulatory modules controlled by the switch, and (b) an increase in the transcriptomic heterogeneity in one of these regulatory modules. The latter may be caused by each of the remaining mechanisms.
- Structural change in a regulator of gene expression levels. DNA copy number alterations are a key example of such changes.
- Temporary change in a regulator of gene expression levels. In particular, the more upstream in a pathway the temporary change occurs, the stronger the transcriptomic entropy increase. For instance, stress induced by exposure to radiation may cause a short-term change in the regulatory system.
- Weakened conditional dependencies between the genes in a pathway. Pathway inactivation serves as an illustration.

In the above mechanisms, hub genes of the regulatory systems play an important role. For example, their inactivation is likely to cause a higher transcriptomic heterogeneity increase than that of more peripheral genes.

Cancer is a complex disease, which exploits many routes to derail the regulatory system and may lead to a transcriptomic heterogeneity increase. The few explanations of dysregulation in the cancer cell via entropy surge offered here are unlikely to be exhaustive. Other mechanisms may exist and need to be identified in order to understand the ways of the cancer cell. Furthermore, all mechanisms need evaluation in the face of nonnormality and nonlinearity. Future research should also concentrate on possible attenuations of the identified mechanisms. For instance, several DNA copy number aberration types, e.g., loss and gain, are often distinguished, and they may have a different effect on the transcriptomic heterogeneity. Finally, it should be noted that the models employed here assume the regulatory system to be isolated, while in fact it operates within the larger environment of the cell, the tissue, and the organism.

## Electronic supplementary material


**Additional Files** Supplementary Material: contains proofs of propositions and other claims; details of used data sets; extensive description of simulations; additional plots. (pdf 361 KB)

## References

[CR1] Band AM, Laiho M (2011). Crosstalk of tgf-$$\beta $$ and estrogen receptor signaling in breast cancer. J. Mammary Gland Biol Neoplasia.

[CR2] Banerji CRS, Miranda-Saavedra D, Severini S, Widschwendter M, Enver T, Zhou JX, Teschendorff AE (2013) Cellular network entropy as the energy potential in Waddington’s differentiation landscape. Sci Rep 3 10.1038/srep03039 PMC3807110 24154593

[CR3] Banerji CRS, Severini S, Caldas C, Teschendorff AE, Tanay A (2015). Intra-tumour signalling entropy determines clinical outcome in breast and lung cancer. PLoS Comput Biol.

[CR4] Berretta R, Moscato P (2010). Cancer biomarker discovery: the entropic hallmark. PLoS One.

[CR5] Björnström L, Sjöberg M (2005). Mechanisms of estrogen receptor signaling: convergence of genomic and nongenomic actions on target genes. Mol Endocrinol.

[CR6] Dang CV, Resar LMS, Emisona E, Kim S, Li Q, Prescott JE, Wonsey D, Zeller K (1999). Function of the c-Myc oncogenic transcription factor. Exp Cell Res.

[CR7] Demetrius L, Gundlach VM, Ochs G (2004). Complexity and demographic stability in population models. Theor Popul Biol.

[CR8] Dexter DL, Leith JT (1986). Tumor heterogeneity and drug resistance. J Clin Oncol.

[CR9] Futreal PA, Coin L, Marshall M, Down T, Hubbard T, Wooster R, Rahman N, Stratton MR (2004). A census of human cancer genes. Nat Rev Cancer.

[CR10] Goldie JH, Coldman AJ (1978) A mathematic model for relating the drug sensitivity of tumors to their spontaneous mutation rate. Cancer Treat Rep 63(11–12):1727–1733 526911

[CR11] Hanahan D, Weinberg R (2000). The hallmarks of cancer. Cell.

[CR12] Hart IR, Fidler IJ (1981). The implications of tumor heterogeneity for studies on the biology and therapy of cancer metastasis. Biochimica et Biophysica Acta.

[CR13] Harville DA (2008). Matrix algebra from a statistician’s perspective.

[CR14] Heppner GH, Miller BE (1983). Tumor heterogeneity: biological implications and therapeutic consequences. Cancer Metastasis Rev.

[CR15] Jonsson PF, Bates PA (2006). Global topological features of cancer proteins in the human interactome. Bioinformatics.

[CR16] Kaila VRI, Annila A (2008). Natural selection for least action. Proc R Soc A.

[CR17] Kauffman SA (1993). Origins of order: self-organization and selection in evolution.

[CR18] Ledoit O, Wolf M (2004). A well conditioned estimator for large-dimensional covariance matrices. J Multivar Anal.

[CR19] Look AT (1997). Oncogenic transcription factors in the human acute leukemias. Science.

[CR20] Marusyk A, Polyak K (2010). Tumor heterogeneity: causes and consequences. Biochimica et Biophysica Acta.

[CR21] Nowell PC (1976). The clonal evolution of tumor cell populations. Science.

[CR22] Ogata H, Goto S, Sato K, Fujibuchi W, Bono H, Kanehisa M (1999). Kegg: kyoto encyclopedia of genes and genomes. Nucleic acids Res.

[CR23] Peng J, Wang P, Zhou N, Zhu J (2009). Partial correlation estimation by joint sparse regression models. J Am Stat Assoc.

[CR24] Pinto CA, Widodo E, Waltham M, Thompson EW (2013). Breast cancer stem cells and epithelial mesenchymal plasticity—implications for chemoresistance. Cancer Lett.

[CR25] Rajapakse I, Groudine M, Meshabi M (2011). Dynamics and control of state-dependent networks for probing genomic organization. PNAS.

[CR26] Ron E, Lubin JH, Shore RE, Mabuchi K, Modan B, Pottern LM, Schneider AB, Tucker MA, Boice JD (1995). Thyroid cancer after exposure to external radiation: a pooled analysis of seven studies. Radiat Res.

[CR27] Schäfer J, Strimmer K (2005) A shrinkage approach to large-scale covariance matrix estimation and implications for functional genomics. Stat Appl Genet Mol Biol **4**, Article 32 10.2202/1544-6115.1175 16646851

[CR28] Schramm G, Kannabiran N, König R (2010). Regulation patterns in signaling networks of cancer. BMC Syst Biol.

[CR29] Schröder M, Haibe-Kains B, Culhane A, Sotiriou C, Bontempi G, J., (2011) Q.: breastCancerMAINZ; breastCancerTRANSBIG;breastCancerUNT; breastCancerUPP; breastCancerVDX. R packages, versions 1.0.6

[CR30] Shackleton M, Quintana E, Fearon ER, Morrison SJ (2009). Heterogeneity in cancer: cancer stem cells versus clonal evolution. Cell.

[CR31] Stingl J, Caldas C (2007). Molecular heterogeneity of breast carcinomas and the cancer stem cell hypothesis. Nat Rev Cancer.

[CR32] Tarabichi M, Antoniou A, Saiselet M, Pita JM, Andry G, Dumont JE, Detours V, Maenhaut C (2013). Systems biology of cancer: entropy, disorder, and selection-driven evolution to independence, invasion and “swarm intelligence”. Cancer Metastasis Rev.

[CR33] Teschendorff AE, Severini S (2010). Increased entropy of signal transduction in the cancer metastasis phenotype. BMC Syst Biol.

[CR34] Teschendorff AE, Sollich P, Kuehn R (2014). Signalling entropy: a novel network-theoretical framework for systems analysis and interpretation of functional omic data. Methods.

[CR35] Van Wieringen WN, Van der Vaart AW (2011). Statistical analysis of the cancer cell’s molecular entropy using high-throughput data. Bioinformatics.

[CR36] Van Wieringen WN, Van de Wiel MA (2014). Penalized differential pathway analysis of integrative oncogenomics studies. Stat Appl Genet Mol Biol.

[CR37] Vogelstein B, Kinzler KW (2004). Cancer genes and the pathways they control. Nat Med.

[CR38] Vogelstein B, Lane D, Levine AJ (2000). Surfing the p53 network. Nature.

[CR39] Wachi S, Yoneda K, Wu R (2005). Interactome-transcriptome analysis reveals the high-centrality of genes differentially expressed in lung cancer tissues. Bioinformatics.

[CR40] Weinberg RA (2006). The biology of cancer.

[CR41] West J, Bianconi G, Severini S, Teschendorff AE (2012) Differential network entropy reveals cancer system hallmarks. Sci Rep 2 10.1038/srep00802 PMC3496163 23150773

[CR42] Zalatan JG, Coyle SM, Rajan S, Sidhu SS, Lim WA (2012). Conformational control of the Ste5 scaffold protein insulates against MAP kinase misactivation. Science.

