# Supplementary material for: Transcriptomic Heterogeneity in Cancer as a Consequence of Dysregulation of the Gene–Gene Interaction Network
Source: Bull Math Biol. 2015 Sep 16;77(9):1768–86. doi: 10.1007/s11538-015-0103-7 (PMC4644214; doi:10.1007/s11538-015-0103-7)
Supplement: Supplementary file 1 — Additional Files Supplementary Material: contains proofs of propositions and other claims; details of used data sets; extensive description of simulations; additional plots. (pdf 361 KB) [file 11538_2015_103_MOESM1_ESM.pdf]

Supplementary Material to: Cancer heterogeneity as a consequence of dysregulation of the gene-gene interaction network

Wessel N. van Wieringen[1,2,*], **Aad W. van der Vaart**[3]

[1] Department of Epidemiology and Biostatistics, VU University Medical Center

P.O. Box 7057, 1007 MB Amsterdam, The Netherlands

[2] Department of Mathematics, VU University Amsterdam

De Boelelaan 1081a, 1081 HV Amsterdam, The Netherlands

[3] Department of Mathematics, Leiden University

P.O. Box 9512, 2300 RA Leiden, The Netherlands

[*]Corresponding author. Email: w.vanwieringen@vumc.nl

SM A: Proof of Proposition 1

Proposition 1. *Let* $\mathbf{Y}$ *be a* p*-variate random variable in* $\mathbb{R}^p$*,* $f_z(\mathbf{Y}) = f(\mathbf{Y}|z)$ *be a density for every* z *in a domain* D*. Then, if* G *is a probability distribution on* D *and* $f_G(\mathbf{Y}) = \int_D f(\cdot|z)\, dG(z)$*:*

$$-\int_{\mathbb{R}^p} f_G(\mathbf{Y}) \log[f_G(\mathbf{Y})]\, d\mathbf{Y} \quad \geq \quad -\int_D \int_{\mathbb{R}^p} f_z(\mathbf{Y}) \log[f_z(\mathbf{Y})]\, d\mathbf{Y}\, dG(z).$$

Proof. It suffices to apply Jensens inequality to the convex function $f \mapsto f \log(f)$ in order to see that $(Ef_Z)\log(Ef_Z) \leq E[f_Z \log(f_Z)]$, whence:

$$-\int_{\mathbb{R}^p} f_G(\mathbf{Y}) \log[f_G(\mathbf{Y})]\, d\mathbf{Y} \quad \geq \quad -\int_{\mathbb{R}^p} \int_D f_z(\mathbf{Y}) \log[f_z(\mathbf{Y})]\, dG(z)\, d\mathbf{Y}. \tag{1}$$

Finally, apply Fubini's theorem. □

SM B: Data set details

Table 1: Details of the five breast data set with gene expression data only.

Name	:	MAINZ, TRANSBIG, UNT, UPP, VDX
Tissue	:	Breast
# samples	:	200 (MAINZ), 198 (TRANSBIG), 126 (UNT), 247 (UPP), 344 (VDX)
		(sample sizes after removal of samples with unknown ER status)
# ER- samples	:	38 (MAINZ), 64 (TRANSBIG), 40 (UNT), 34 (UPP), 135 (VDX)
# ER+ samples	:	162 (MAINZ), 134 (TRANSBIG), 86 (UNT), 213 (UPP), 209 (VDX)
GE platform	:	Affymetrix HGU133a (MAINZ, TRANSBIG, VDX),
		Affymetrix HGU133ab (UNT, UPP)
Preprocessing GE	:	Detailed in the R-packages `breastCancerMAINZ`,
	:	`breastCancerTRANSBIG`, `breastCancerUNT`, `breastCancerUPP`,
	:	`breastCancerVDX`.
# GE features (NOTCH)	:	81 (MAINZ), 81 (TRANSBIG), 115 (UNT), 115 (UPP), 81 (VDX)
		(pathway defined by KEGG using the `KEGG.db` package)
# GE features (TGFβ)	:	142 (MAINZ), 142 (TRANSBIG), 194 (UNT), 194 (UPP), 142 (VDX)
		(pathway defined by KEGG using the `KEGG.db` package)
Availibility GE	:	R-packages `breastCancerMAINZ`, `breastCancerTRANSBIG`,
	:	`breastCancerUNT`, `breastCancerUPP`, `breastCancerVDX`

Table 2: Details of the Chin breast cancer data set with both DNA copy number and gene expression data.

Name	:	Chin
Tissue	:	Breast
# samples	:	89
# ER- samples	:	34
# ER+ samples	:	55
GE platform	:	Affymetrix HGU133A
CN platform	:	OncoBAC arrays
Citation	:	Chin *et al.* (2006)
Preprocessing GE	:	As detailed in Klijn *et al.* (2008) and Van Wieringen *et al.* (2012). In addition, features that could not be mapped to a genomic location are removed.
Preprocessing CN	:	As detailed in Klijn *et al.* (2008) and Van Wieringen *et al.* (2012). In addition, features that could not be mapped to a genomic location are removed.
Matching CN-GE	:	Chromosomal location (start and end base pair) of features of both platforms are obtained. Each feature in the expression data set is matched to the feature from the copy number platform with the maximum percentage of genomic overlap. If the maximum percentage of overlap equals zero, the gene is not included in the matched objects. This procedure is implemented in the R-package `sigaR`, available from Bioconductor and described in detail in Van Wieringen *et al.* (2012).
# GE features (NOTCH)	:	33 (pathway defined by KEGG using the `KEGG.db` package)
# CN features (NOTCH)	:	33 (equal to # GE features due to matching)
# GE features (TGFβ)	:	64 (pathway defined by KEGG using the `KEGG.db` package)
# CN features (TGFβ)	:	64 (equal to # GE features due to matching)
Availibility GE	:	ArrayExpress (accession number: E-TABM-158).
Availibility CN	:	`http://cancer.lbl.gov/data.htm`

Table 3: Details of the NKI breast cancer data set with both DNA copy number and gene expression data.

Name	:	NKI
Tissue	:	Breast
# samples	:	68
# ER- samples	:	25
# ER+ samples	:	43
GE platform	:	Rosetta
CN platform	:	Array CGH
Citation	:	Horlings *et al.* (2010)
Preprocessing GE	:	Detailed in package `breastCancerNKI`.
Preprocessing CN	:	The $\log_2$ ratios are median normalized. In addition, features that could not be mapped to a genomic location are removed.
Matching CN-GE	:	Chromosomal location (start and end base pair) of features of both platforms are obtained. Each feature in the expression data set is matched to the feature from the copy number platform with the maximum percentage of genomic overlap. If the maximum percentage of overlap equals zero, the gene is not included in the matched objects. This procedure is implemented in the R-package `sigaR`, available from Bioconductor and described in detail in Van Wieringen *et al.* (2012).
# GE features (NOTCH)	:	32
# CN features (NOTCH)	:	32 (equal to # GE features due to matching)
# GE features (TGFβ)	:	58
# CN features (TGFbβ)	:	58 (equal to # GE features due to matching)
Availibility GE	:	R-packages `breastCancerNKI`
Availibility CN	:	Data provided as Supplementary Data File S1, log 2 aCGH data, to the article (Horlings *et al.*, 2010).
Remark	:	The data set is limited to only those samples for which both CN and GE data is available.

Table 4: Details of the Zhang breast cancer data set with both DNA copy number and gene expression data.

Name	:	Zhang
Tissue	:	Breast
# samples	:	263
# ER- samples	:	69
# ER+ samples	:	194
GE platform	:	Affymetrix HGU133a
CN platform	:	Affymetrix GeneChip Human Mapping 100k
Citation	:	Wang *et al.* (2005); Zhang *et al.* (2009)
Preprocessing GE	:	Detailed in Wang *et al.* (2005). In addition, genes that could not be mapped to a chromosomal location are removed, and expression of features that map (exactly) to the same location is averaged.
Preprocessing CN	:	After removal of bad SNPs (determined by default settings of the Affymetrix Genotyping software), the $\log_2$ ratios are median normalized. In addition, features that could not be mapped to a genomic location are removed.
Matching CN-GE	:	The features of both platforms are matched by their percentage of overlap (see Table 9 for details).
# GE features (NOTCH)	:	49 (pathway defined by KEGG using the `KEGG.db` package)
# CN features (NOTCH)	:	49 (equal to # GE features due to matching)
# GE features (TGFβ)	:	98 (pathway defined by KEGG using the `KEGG.db` package)
# CN features (TGFβ)	:	98 (equal to # GE features due to matching)
Availibility GE	:	GEO (username: jyu8; password: jackxyu)
Availibility CN	:	GEO (GSE10099)
Remark	:	The data set is limited to only those samples for which both CN and GE data is available.

SM C: Simulation, Section 3

Here we investigate – by simulation – whether a node's connectivity affects the positive concordant relationship between genomic and transcriptomic entropy within a pathway. Starting point is the simultaneous-equations model derived in the main text from the rate equations:

$$\mathbf{\Theta} \mathbf{Y} \,|\, \mathbf{X} \quad = \quad \boldsymbol{\beta} \circ \mathbf{X} + \boldsymbol{\varepsilon},$$

which relates the expression of each gene to its own DNA copy number and to the expression levels of the other genes in the pathway. Given a regulatory network (parametrized by $\mathbf{\Theta}$) and the DNA copy number effects ($\boldsymbol{\beta}$) on expression levels, the genomic entropy (reflected in $\mathbf{X}$) of each gene is increased (one at a times) and the effect on the entropy of the network studied within this simulation.

First we describe how the regulatory network is sampled, which can either have a scale-free or small world network topology. These topologies are generated in accordance with the procedures of either Barabási and Albert (1999) or Watts and Strogatz (1998). We employ the implementation of these procedures in the R package `igraph` (Csardi and Nepusz, 2006). The scale free network is generated with parameters $m = 2$ (the number of edges added in each step), and the probability of an old node being connected to the new set proportional to $k^{-\gamma} + a_0$. In this expression k is the node's degree, $\gamma = 0.7$ is the power, and $a_0 = 3$ is an off-set to increase the chances of a low connected node being selected. The small world network is generated from a lattice of p nodes each connected to two neighbors ($nei = 2$), after which there is a 50% chance of each edge being rewired. Only undirected networks are generated. It may happen that a generated network is not connected. This is then discarded and a new network is generated until we obtain a connected one. Having obtained the network topology, $\mathbf{\Theta}$ is set equal to the adjacency matrix of the network. Each off-diagonal, non-zero element of $\mathbf{\Theta}$ is replaced by either 0.3 or -0.3, randomly chosen by an unbiased coin.

Having specified $\mathbf{\Theta}$, the remaining parameters of the model are set as follows. The DNA copy number effect on the expression levels is set equal to one for all genes, i.e. $\boldsymbol{\beta} = \mathbf{1}_{p\times 1}$. Finally, the variance of the error $\boldsymbol{\varepsilon}$ is taken to be $\mathbf{\Sigma} = 0.3\,\mathbf{I}$.

In order to see the effect of an increase in genomic entropy most clearly, we allow only one gene at the time to have a DNA copy number effect on the transcription levels. Hereto the variance of the DNA copy number $\text{Var}(\mathbf{X})$ is set equal to $\mathbf{0}_{pp}$, with the j-th diagonal element equal to 1. All off-diagonal elements are also zero.

Given the network topology (as conveyed by $\mathbf{\Theta}$), we calculate the edge degree, d_j, of node j and increase its genomic entropy by setting the j-th diagonal element of $\text{Var}(\mathbf{X})$ equal to one. The resulting transcriptomic entropy of the pathway, denoted H_j, is simply $\log\{\det[\text{Var}(\mathbf{Y})]\}$, where $\text{Var}(\mathbf{Y})$ is given in the main text and index j refers to the node with increased genomic entropy. This is done for each node $j = 1, \ldots, p$. Finally, the relationship between d_j is H_j is visualized (Figure 1).

Figure 1 clearly shows that the transcriptomic entropy increases (all y-axes start at a positive value). More importantly, Figure 1 reveals that the transcriptomic entropy increase is unrelated to the degree of the affected node (as the horizontal lines imply).

The absence of a relation between a node's connectivity and transcriptomic entropy increase can be understood analytically. Consider the situation where node j is affected. The variance of $\mathbf{Y}$ is then:

$$\text{Var}(\mathbf{Y}) \quad = \quad \mathbf{\Theta}^{-1}(\mathbf{e}_j \mathbf{e}_j^{\mathrm{T}} + \mathbf{\Sigma})(\mathbf{\Theta}^{-1})^{\mathrm{T}},$$

where $\mathbf{e}_j$ is a unit vector with a one at the j-th position and zero's at the remaining $p-1$ position.

Then,

$$
\begin{aligned}
|\mathrm{Var}(\mathbf{Y})| &= (|\mathbf{\Theta}|)^{-2}\,|(\mathbf{e}_j\mathbf{e}_j^{\mathrm{T}} + \mathbf{\Sigma})| \\
&= (|\mathbf{\Theta}|)^{-2}\,|\mathbf{\Sigma}|\,(1 + \mathbf{e}_j^{\mathrm{T}}\mathbf{\Sigma}^{-1}\mathbf{e}_j), \\
&= (|\mathbf{\Theta}|)^{-2}\,|\mathbf{\Sigma}|\,[1 + (\mathbf{\Sigma}^{-1})_{jj}],
\end{aligned}
$$

where we have used the Matrix Determinant lemma. As the nodes have the same residual variance, the effect of increasing the genomic entropy is identical for each node. Consequently, the transcriptomic entropy increase is unrelated to the degree of the affected node.

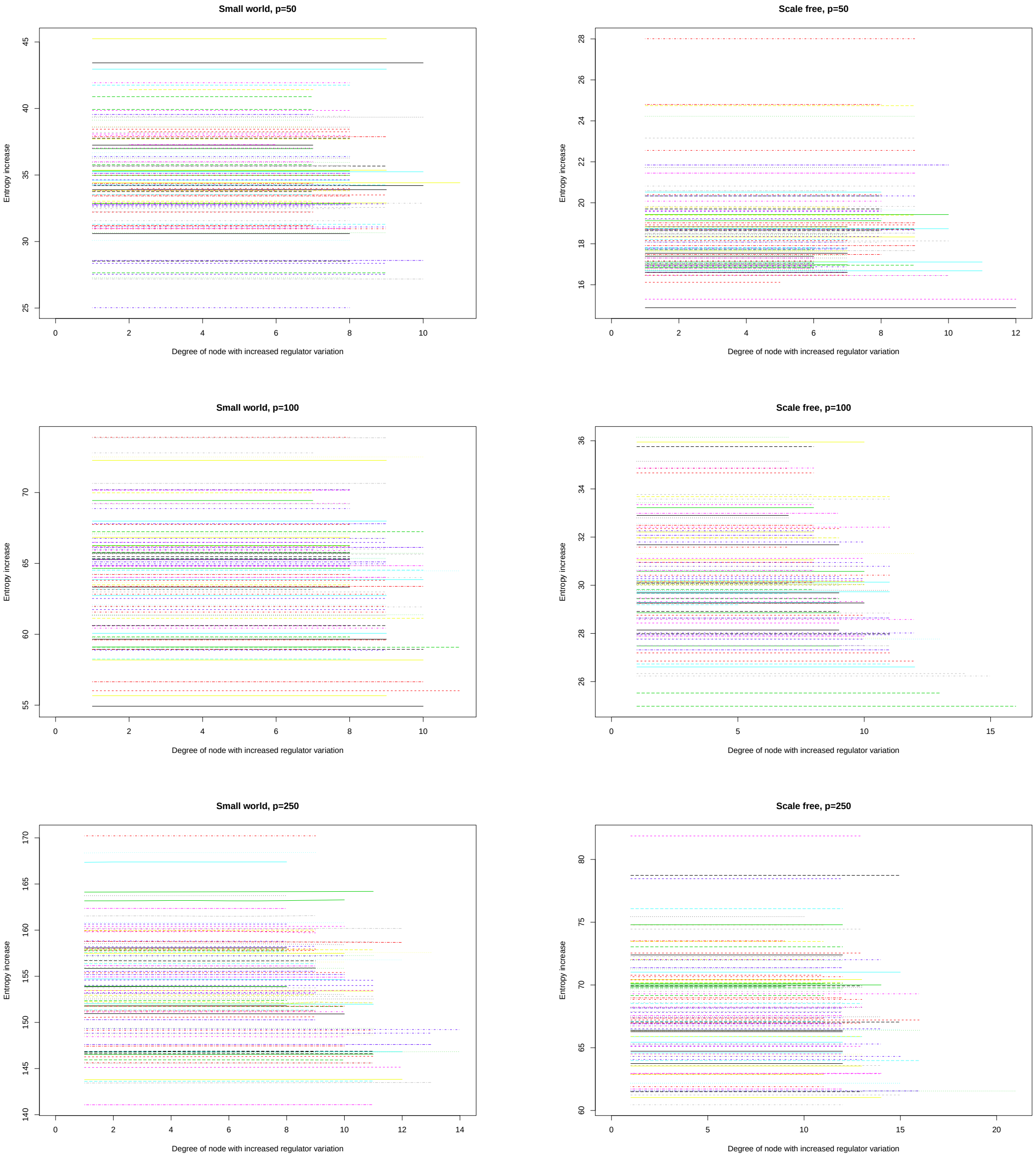

Figure 1: Each panel shows cubic spline smoothed relationship between the node degree of the node with increased genomic entropy vs. the corresponding transcriptomic entropy. Each panel depicts a hundred instances of the relationship. In the left (right) panels a small world (scale free) topology underlies the pathway. Different pathway sizes are considered, from top to bottom: $p = 50, 100, 250$.

SM D: Entropy of genomic and transcriptomic level

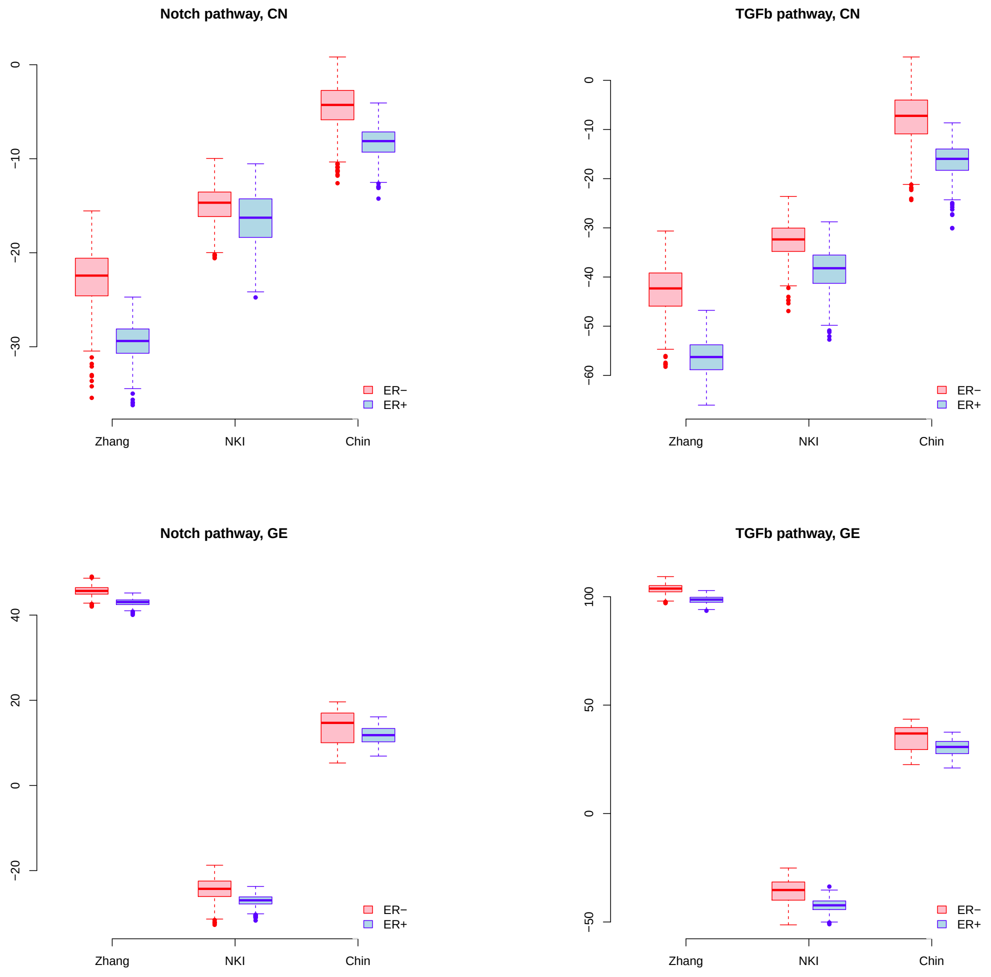

Figure 2: Boxplots of entropy estimates versus ER status of three breast cancer studies. The top and bottom panels display the entropy of at the genomic and transcriptomic level, respectively.

SM E: Univariate gene dosage variance and effect

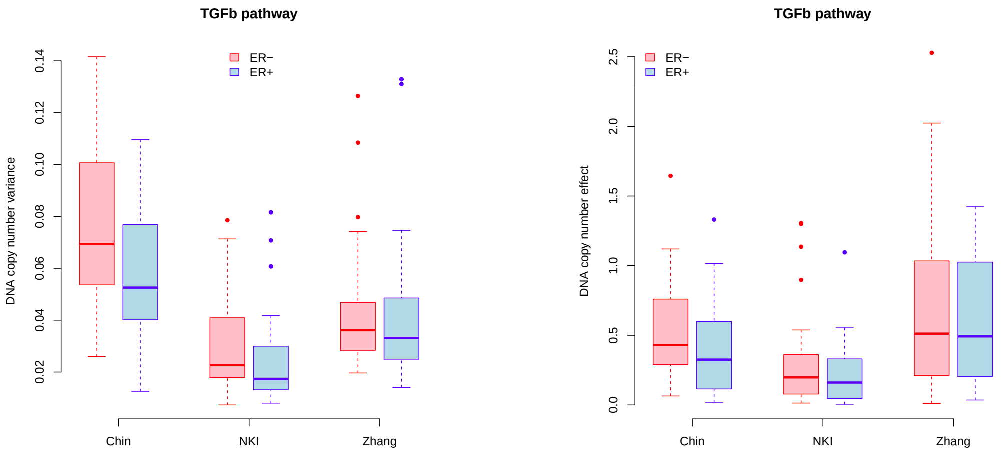

Figure 3: Variance of DNA copy number (left panel) and its estimated effect on gene expression (right) in the TGFβ pathway

SM F: Disturbance in VAR(1) illustration

The VAR(1) model used to generate the Figure 5 is parametrized by:

$$\nu = \begin{pmatrix} 0 \\ 0 \\ 0 \end{pmatrix}, \quad \mathbf{A} = \begin{pmatrix} 3/4 & 0 & 0 \\ -5/2 & 0 & 0 \\ 9/5 & -3/2 & 2/5 \end{pmatrix} \quad \text{and} \quad \mathbf{\Sigma}_\varepsilon = \begin{pmatrix} 1/4 & 0 & 0 \\ 0 & 1/4 & 0 \\ 0 & 0 & 1/4 \end{pmatrix}^{-1}.$$

From this model, a time series of length 50 is generated. For the perturbed sequence a disturbance $\boldsymbol{\delta}_t = (X, 0, 0)^{\mathrm{T}}$, where X is sampled from $\mathcal{N}(0, 4)$, is added at time point 25. Apart from this time point, the innovations of both sequences are identical. Only the data from the first variate of both generated sequences are plotted.

SM G: Proof of proposition 2

Assume the vector of gene expressions at time point t, denoted $\mathbf{Y}_t$, can be modeled by a VAR(1) (first-order vector autoregressive) process:

$$\mathbf{Y}_t \quad = \quad \boldsymbol{\nu} + \mathbf{A}\mathbf{Y}_{t-1} + \boldsymbol{\varepsilon}_t, \qquad (2)$$

where $\boldsymbol{\nu}$ the $p \times 1$ intercept vector, $\mathbf{A}$ a $p \times p$ coefficient matrix, and $\boldsymbol{\varepsilon}_t$ a $p \times 1$ vector with the errors. It is assumed that $\boldsymbol{\varepsilon}_t \sim \mathcal{N}(\mathbf{0}_{p\times 1}, \boldsymbol{\Sigma}_\varepsilon)$ and $\text{Cov}(\boldsymbol{\varepsilon}_{t_1}, \boldsymbol{\varepsilon}_{t_2}) = \mathbf{0}$ if $t_1 \neq t_2$. Introduction of a disturbance at time point τ modifies model (2) to:

$$\mathbf{Y}_t^{(\tau)} \quad = \quad \boldsymbol{\nu} + \mathbf{A}\mathbf{Y}_{t-1} + \boldsymbol{\varepsilon}_t + \boldsymbol{\delta}_\tau I_{\{t=\tau\}}, \qquad (3)$$

where $\boldsymbol{\delta}_\tau \sim \mathcal{N}(\mathbf{0}_{p\times 1}, \boldsymbol{\Sigma}_\delta)$ a $p \times 1$ vector with the disturbances. We can now formulate the following proposition:

Proposition 2. *Let $\mathbf{Y}_t$ and $\mathbf{Y}_t^{(\tau)}$ be p-variate random variables distributed in accordance with models (2) and (3). Then, $Var[\mathbf{Y}_t^{(\tau)}] \succ Var(\mathbf{Y}_t)$ and $Var[\mathbf{Y}_t^{(\tau_1)}] \succ Var[\mathbf{Y}_t^{(\tau_2)}]$ if $\tau_2 > \tau_1$.*

Proof. Iterative application of formula (2) gives:

$$\mathbf{Y}_t^{(\tau)} \quad = \quad \sum_{s=1}^{t} \mathbf{A}^{t-s}\boldsymbol{\nu} + \sum_{s=1}^{t} \mathbf{A}^{t-s}\boldsymbol{\varepsilon}_s + \sum_{s=\tau}^{t} \mathbf{A}^{t-s}\boldsymbol{\delta}_\tau.$$

Hence, the variance of $\mathbf{Y}_t^{(\tau)}$:

$$\text{Var}[\mathbf{Y}_t^{(\tau)}] \quad = \quad \sum_{s=1}^{t} \mathbf{A}^{t-s}\boldsymbol{\Sigma}_\varepsilon[\mathbf{A}^{t-s}]^{\mathrm{T}} + \sum_{s=\tau_0}^{t} \mathbf{A}^{t-s}\boldsymbol{\Sigma}_\delta[\mathbf{A}^{t-s}]^{\mathrm{T}}.$$

As the second summand on the right-hand side is positive definite, we obtain:

$$\begin{aligned}
\text{Var}(\mathbf{Y}_t^{(\tau_0)}) \quad &= \quad \sum_{s=1}^{t} \mathbf{A}^{t-s}\boldsymbol{\Sigma}_\varepsilon[\mathbf{A}^{t-s}]^{\mathrm{T}} + \sum_{s=\tau_0}^{t} \mathbf{A}^{t-s}\boldsymbol{\Sigma}_\delta[\mathbf{A}^{t-s}]^{\mathrm{T}} \\
&\succ \quad \sum_{s=1}^{t} \mathbf{A}^{t-s}\boldsymbol{\Sigma}_\varepsilon[\mathbf{A}^{t-s}]^{\mathrm{T}} \quad = \quad \text{Var}(\mathbf{Y}_t).
\end{aligned}$$

This proves the first assertion.

Now if $\tau_1 < \tau_2$, then:

$$\begin{aligned}
\text{Var}(\mathbf{Y}_t^{(\tau_1)}) \quad &= \quad \sum_{s=1}^{t} \mathbf{A}^{t-s}\boldsymbol{\Sigma}_\varepsilon[\mathbf{A}^{t-s}]^{\mathrm{T}} + \sum_{s=\tau_1}^{t} \mathbf{A}^{t-s}\boldsymbol{\Sigma}_\delta[\mathbf{A}^{t-s}]^{\mathrm{T}} \\
&= \quad \sum_{s=1}^{t} \mathbf{A}^{t-s}\boldsymbol{\Sigma}_\varepsilon[\mathbf{A}^{t-s}]^{\mathrm{T}} + \sum_{s=\tau_2}^{t} \mathbf{A}^{t-s}\boldsymbol{\Sigma}_\delta[\mathbf{A}^{t-s}]^{\mathrm{T}} + \sum_{s=\tau_1}^{\tau_2-1} \mathbf{A}^{t-s}\boldsymbol{\Sigma}_\delta[\mathbf{A}^{t-s}]^{\mathrm{T}} \\
&\succ \quad \sum_{s=1}^{t} \mathbf{A}^{t-s}\boldsymbol{\Sigma}_\varepsilon[\mathbf{A}^{t-s}]^{\mathrm{T}} + \sum_{s=\tau_2}^{t} \mathbf{A}^{t-s}\boldsymbol{\Sigma}_\delta[\mathbf{A}^{t-s}]^{\mathrm{T}} \quad = \quad \text{Var}(\mathbf{Y}_t^{(\tau_2)}).
\end{aligned}$$

Hence, also the second assertion is proven. □

SM H: Proof of Proposition 3

Proposition 3. *Let $\mathbf{X} \sim \mathcal{N}(\mathbf{0}, \mathbf{\Sigma}_X)$ and $\mathbf{Y} \sim \mathcal{N}(\mathbf{0}, \mathbf{\Sigma}_Y)$ with equal marginal variances diag($\mathbf{\Sigma}_X$) = diag($\mathbf{\Sigma}_Y$). Further, assume that the $p \times p$ partial correlation matrices (i.e. concentration matrices standardized to have a unit diagonal) associated with $\mathbf{X}$ and $\mathbf{Y}$, denoted $\mathbf{\Omega}_{\boldsymbol{\gamma}^{(x)}}$ and $\mathbf{\Omega}_{\boldsymbol{\gamma}^{(y)}}$ can be both partitioned as $r \times r$ block matrices:*

$$\mathbf{\Omega}_{\boldsymbol{\gamma}} = \begin{pmatrix} \mathbf{\Omega}_{11} & \gamma_{12}\mathbf{\Omega}_{12} & \dots & \gamma_{1r}\mathbf{\Omega}_{1r} \\ \gamma_{12}\mathbf{\Omega}_{12}^T & \mathbf{\Omega}_{22} & & \vdots \\ \vdots & & \ddots & \vdots \\ \gamma_{1r}\mathbf{\Omega}_{1r}^T & \dots & \dots & \mathbf{\Omega}_{rr} \end{pmatrix},$$

with $\boldsymbol{\gamma} = (\gamma_{12}, \dots, \gamma_{1r}, \gamma_{23}, \dots, \gamma_{2r}, \dots, \gamma_{r-1,r}) \in [0,1]^{\frac{1}{2}r(r-1)}$. Then, $\boldsymbol{\gamma}^{(x)} \leq \boldsymbol{\gamma}^{(y)}$ (element-wise) implies $H(\mathbf{Y}) \leq H(\mathbf{X})$.

Proof. Since we can repeat the values of γ_{ij} it is not a loss of generality to assume that the block sizes are equal to 1. Furthermore, because we can apply the inequality repeatedly coordinatewise, it suffices to consider $\gamma^{(x)} \leq \gamma^{(y)}$ that differ in one coordinate only, which then can be taken to be the last one, as otherwise the vector can be reordered.

A random vector $X = (X_1, \dots, X_r)^T$ can be represented as

$$\begin{pmatrix} X_1 \\ X_2 \\ \vdots \\ X_r \end{pmatrix} = \begin{pmatrix} I & 0 & \cdots & 0 & 0 \\ P_{2,1} & I & \cdots & 0 & 0 \\ \vdots & & & & \\ P_{r,1} & P_{r,2} & \cdots & P_{r,r-1} & I \end{pmatrix} \begin{pmatrix} R_1 \\ R_2 \\ \vdots \\ R_r \end{pmatrix}, \tag{4}$$

where $R_1 = X_1$ and R_i is the residual of the linear regression of X_i onto $X_1, \dots, X_{i-1}$ for $i = 2, \dots, r$. Because the residuals $R_1, \dots, R_r$ are uncorrelated and their cumulative spans are the same as those of $X_1, \dots, X_r$, we have for $Q_1, \dots, Q_r$ their covariance matrices,

$$\begin{aligned} \text{Cov}(X_i) &= \sum_{j=1}^{i-1} P_{i,j} Q_j P_{i,j}^T + Q_i, & (5) \\ \text{Cov}(X_{r-1} | X_1, \dots, X_{r-2}) &= Q_{r-1}, & (6) \\ \text{Cov}(X_r, X_{r-1} | X_1, \dots, X_{r-2}) &= P_{r,r-1} Q_{r-1}, & (7) \\ \text{Cov}(X_r | X_1, \dots, X_{r-2}) &= P_{r,r-1} Q_{r-1} P_{r,r-1}^T + Q_r. & (8) \end{aligned}$$

The conditional correlation, given in the $(r-1, r)$-entry of the precision matrix, is the quotient of (7) divided by the product of the roots of (6) and (8).

Now form another vector X^γ by keeping $R_1, \dots, R_{r-1}$ and $P_{i,j}$ for $i < r$ and $P_{r,j}$ for $j < r-1$ in (4) the same, but making the substitutions:

- $P_{r,r-1} \longrightarrow P_{r,r-1}^\gamma := \gamma P_{r,r-1}$,
- $R_r \longrightarrow R_r^\gamma$, orthogonal to $R_1, \dots, R_{r-1}$ and with covariance matrix $Q_r^\gamma := Q_r + (1 - \gamma^2) P_{r,r-1} Q_{r-1} P_{r,r-1}^T$.

The right hand side of (5) remains algebraically identical for $i = 1, \dots, r-1$, and its value for $i = r$ also does not change under this substitution, in view of the definition of Q_r^γ. The same is true for the right hand sides of (6) and (8). Only the right hand side of (7) changes, by a multiplication by γ. Thus the coordinates of the new vector X^γ possess the same variances, and the conditional

correlation of X^γ_{r-1} and X^γ_r given the other coordinates of X^γ is γ times the same quantity for the original vector X.

Because the matrix in (4) is triangular with ones on the diagonal, the determinant of the covariance matrix of X is the same as the determinant of the covariance matrix of $(R_1, \ldots, R_r)^T$, which is $\prod_{i=1}^{r} \det Q_i$. Under the substitution $Q_r \longrightarrow Q^\gamma_r$, the latter product becomes bigger, as $Q^\gamma_r \geq Q_r$.

This proves the assertion, as we can define γ as the quotient of $\gamma^{(x)}$ and $\gamma^{(y)}$. □

SM I: Marginal dependencies

Proposition 4. *Suppose that a symmetric, positive definite matrix* $\mathbf{\Sigma}$ *is partitioned as:*

$$\mathbf{\Sigma} \quad = \quad \begin{pmatrix} \mathbf{\Sigma}_{11} & \mathbf{\Sigma}_{12} \\ \mathbf{\Sigma}_{12}^T & \mathbf{\Sigma}_{22} \end{pmatrix}.$$

For $\gamma \in [0,1]$, *define*

$$\mathbf{\Sigma}_{\gamma} \quad = \quad \begin{pmatrix} \mathbf{\Sigma}_{11} & \gamma\mathbf{\Sigma}_{12} \\ \gamma\mathbf{\Sigma}_{12}^T & \mathbf{\Sigma}_{22} \end{pmatrix}.$$

Then, $|\mathbf{\Sigma}_{\gamma_1}| \leq |\mathbf{\Sigma}_{\gamma_2}|$ *if* $\gamma_1 \geq \gamma_2$.

Proof. From Theorem 13.3.8 of Harville (2008) it is known that $|\mathbf{\Sigma}_{\gamma}| = |\mathbf{\Sigma}_{11}|\,|\mathbf{\Sigma}_{22} - \gamma^2 \mathbf{\Sigma}_{12}^T \mathbf{\Sigma}_{11}^{-1} \mathbf{\Sigma}_{12}|$. Hence, we are left to show that $|\mathbf{\Sigma}_{22} - \gamma_1^2 \mathbf{\Sigma}_{12}^T \mathbf{\Sigma}_{11}^{-1} \mathbf{\Sigma}_{12}| \leq |\mathbf{\Sigma}_{22} - \gamma_2^2 \mathbf{\Sigma}_{12}^T \mathbf{\Sigma}_{11}^{-1} \mathbf{\Sigma}_{12}|$. Now observe that *i)* from Corollary 14.8.6 of Harville (2008) it follows that $\mathbf{\Sigma}_{22} - \gamma^2 \mathbf{\Sigma}_{12}^T \mathbf{\Sigma}_{11}^{-1} \mathbf{\Sigma}_{12} \succ 0$, and *ii)* from Theorem 14.2.9 of Harville (2008) we have that $\gamma^2 \mathbf{\Sigma}_{12}^T \mathbf{\Sigma}_{11}^{-1} \mathbf{\Sigma}_{12} \succeq 0$. The latter, together with $\gamma_1 \geq \gamma_2 \geq 0$, implies $\gamma_1^2 \mathbf{\Sigma}_{12}^T \mathbf{\Sigma}_{11}^{-1} \mathbf{\Sigma}_{12} \succeq \gamma_2^2 \mathbf{\Sigma}_{12}^T \mathbf{\Sigma}_{11}^{-1} \mathbf{\Sigma}_{12} \succeq 0$. This gives:

$$\mathbf{\Sigma}_{22} - \gamma_2^2 \mathbf{\Sigma}_{12}^T \mathbf{\Sigma}_{11}^{-1} \mathbf{\Sigma}_{12} - \mathbf{\Sigma}_{22} + \gamma_1^2 \mathbf{\Sigma}_{12}^T \mathbf{\Sigma}_{11}^{-1} \mathbf{\Sigma}_{12} \quad = \quad (\gamma_1^2 - \gamma_2^2)\, \mathbf{\Sigma}_{12}^T \mathbf{\Sigma}_{11}^{-1} \mathbf{\Sigma}_{12} \quad \succeq \quad 0$$

Application of Corollary 18.1.7 of Harville (2008) now yields the result. □

The above result does not extend to $r \times r$ block matrices when $r \geq 3$. A counter-example is found in the following matrix:

$$\mathbf{\Sigma}_{\gamma} \quad = \quad \begin{pmatrix} 1 & 0.9 & 0.9 \\ 0.9 & 1 & \gamma \\ 0.9 & \gamma & 1 \end{pmatrix}.$$

Then, for $\gamma = 0.70$ $\det(\mathbf{\Sigma}) = 0.024$, while for $\gamma = 0.95$ $\det(\mathbf{\Sigma}) = 0.0165$.

SM J: Simulation II

In the simulation study presented here the effect of connectivity on the entropy is assessed by eliminating dependencies. Starting point of the simulation is a covariance matrix $\mathbf{\Sigma}$ and its associated causal graph (either small world or scale free). For node j we calculate its degree d_j, eliminate its (either marginal or conditional) dependencies with the other nodes and calculate $\log(|\tilde{\mathbf{\Sigma}}^{(j)}|)$ (the entropy), where $\tilde{\mathbf{\Sigma}}$ is obtained from $\mathbf{\Sigma}$ by setting all (either marginal or conditional) dependencies of node j to zero. This is done for each node. Finally, the relationship between d_j is $\log(|\tilde{\mathbf{\Sigma}}^{(j)}|)$ is visualized.

We first describe how we sample the covariance matrices used in the simulation. Either a scale-free or small world network topology is generated in accordance with the procedure of either Barabási and Albert (1999) or Watts and Strogatz (1998), respectively. We employ the implementation of these procedures in the R package `igraph` (Csardi and Nepusz, 2006). The scale free network is generated with parameters $m = 2$ (the number of edges added in each step), and the probability of an old node being connected to the new set proportional to $k^{-\gamma} + a_0$. Here k is the node's degree, $\gamma = 0.7$ the power, and $a_0 = 3$ an off-set to increase the chances of a low connected node being selected. The small world network is generated from a lattice of p nodes each connected to two neighbors ($nei = 2$), after which there is a 50% chance of each edge being rewired. Only undirected networks are generated. It may happen that a generated network is not connected. This is then discarded and a new network is generated until we obtain a connected one. Having obtained the network topology, $\mathbf{\Omega}$ is set equal to the Laplacian of the network. Each upper diagonal, non-zero element of $\mathbf{\Omega}$ is replaced by a random draw from $\mathcal{U}[-1, 1]$. To ensure symmetry of the concentration matrix, the lower diagonal elements of $\mathbf{\Omega}$ are set equal to their upper diagonal counterparts. The diagonal elements of $\mathbf{\Omega}$ are replaced by ones. Finally, $\mathbf{\Sigma} = \mathbf{\Omega}^{-1}$, and $\mathbf{\Sigma}$ is rescaled to have a unit diagonal.

Given the network topology and the associated covariance matrix $\mathbf{\Sigma}$, we calculate the edge degree, d_j, of node j and eliminate its conditional dependencies with the other nodes. For conditional dependency removal, the non-diagonal elements in the j-th row and column of $\mathbf{\Sigma}^{-1}$ are set to zero, after which the covariance matrix with removed conditional dependencies is rescaled to have a unit diagonal again. The resulting covariance matrix is denoted as $\tilde{\mathbf{\Sigma}}^{(j)}$. Next, the entropy associated with the process $\mathcal{N}(\mathbf{0}, \tilde{\mathbf{\Sigma}}^{(j)})$ is calculated: $\log(|\tilde{\mathbf{\Sigma}}^{(j)}|)$. This is done for each node $j = 1, \ldots, p$. Finally, the relationship between d_j is $\log(|\tilde{\mathbf{\Sigma}}^{(j)}|)$ is visualized in Figures 4 and 5.

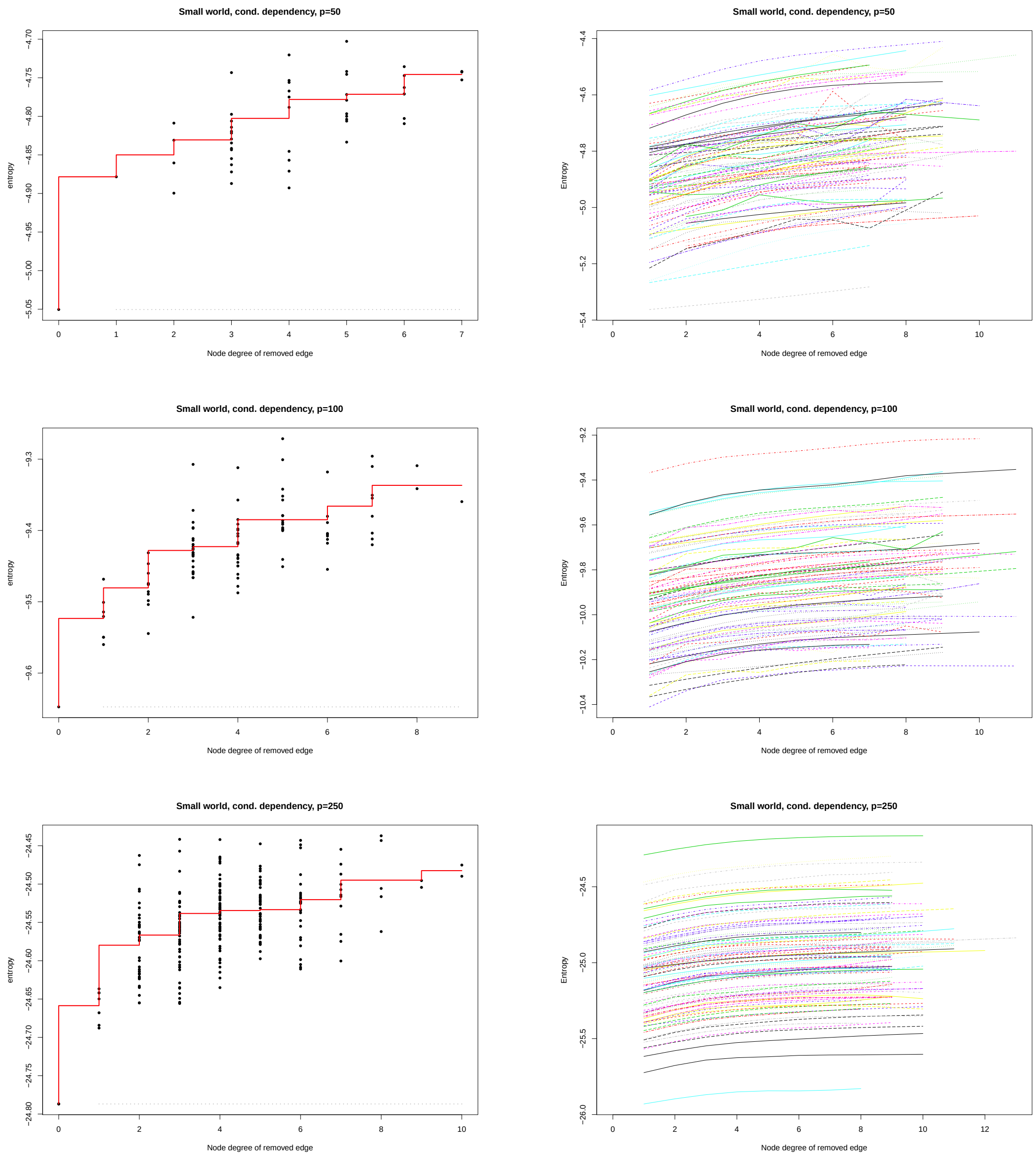

Figure 4: The panels on the left show the node degree of the node with eliminated conditional dependencies vs. the entropy of the resulting covariance matrix with an underlying small world topology (from top to bottom: $p = 50, 100, 250$). The red line is the isotonic regression fit. The panels on the right show cubic spline smoothed relationship between the node degree of the node with eliminated conditional dependencies vs. the entropy of the resulting covariance matrix with an underlying small world topology for hundred instances (from top to bottom: $p = 50, 100, 250$).

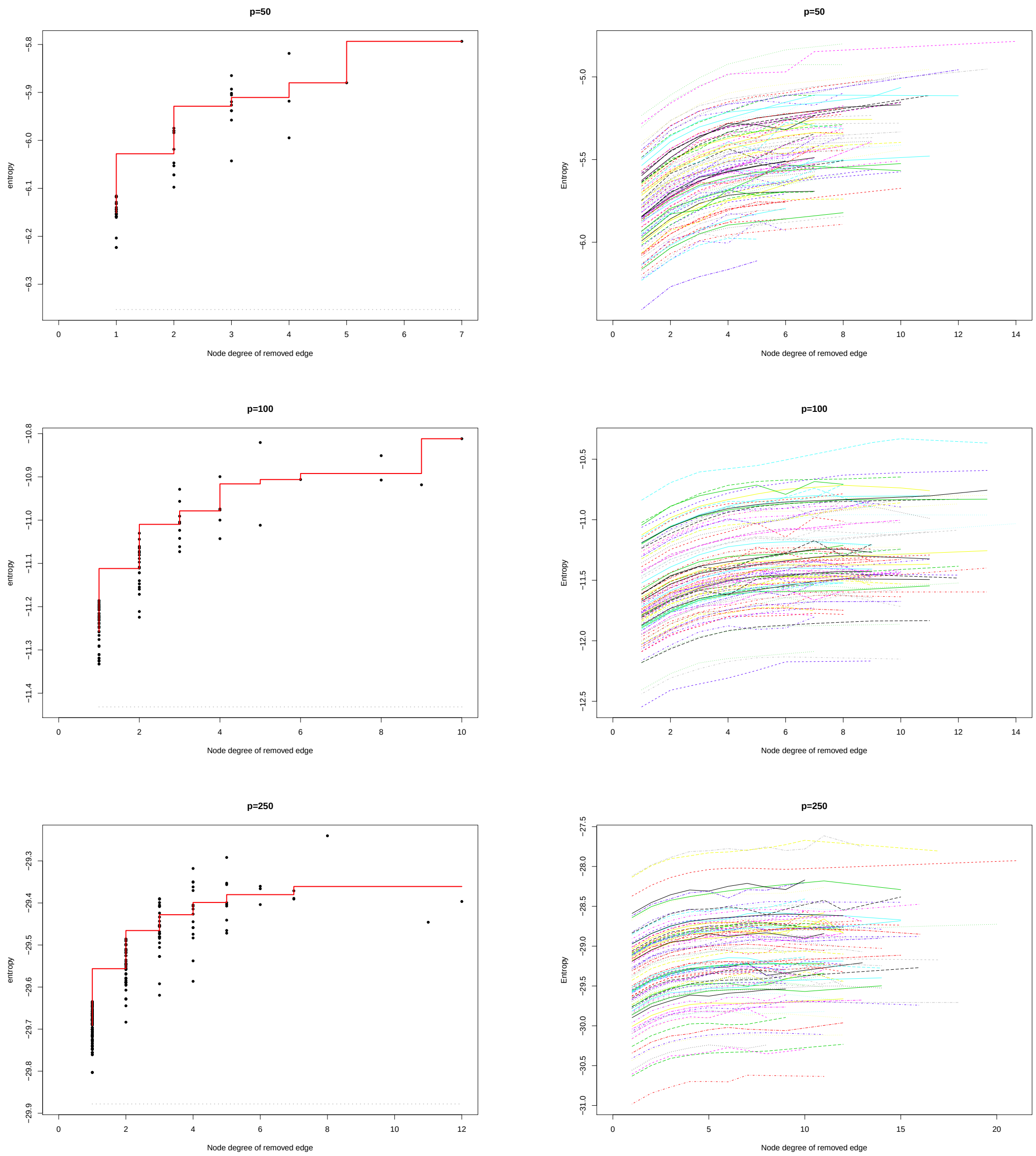

Figure 5: The panels on the left show the node degree of the node with eliminated marginal dependencies vs. the entropy of the resulting covariance matrix with an underlying scale free topology (from top to bottom: $p = 50, 100, 250$). The red line is the isotonic regression fit. The panels on the right show cubic spline smoothed relationship between the node degree of the node with eliminated marginal dependencies vs. the entropy of the resulting covariance matrix with an underlying scale free topology for hundred instances (from top to bottom: $p = 50, 100, 250$).

SM K: Edges vs. penalty parameter, transcriptome only data sets

Does the gene-gene interactions indeed weaken or vanish when shifting from ER+ to ER- status? Hereto we compare the number of conditional dependencies in the Notch and TGFβ pathway, known to be often implicated in breast cancer. This is done in five breast cancerstudies with only gene expression profiles (details in the Appendix B of the SM). The expression data are limited to the genes that map to the pathways (as defined by the KEGG repository). For each pathway data set we subsample repetitively (500 times) an equal number of samples from each ER group. This number of samples is set at 90% of the sample size of the group with the smallest representation in the data set. Now for a given penalty parameter λ_1 the number of edges (number of non-zero partial correlations) in each group is determined using the method of Peng *et al.* (2009). The number of edges found is averaged over the 500 subsamples. The above is repeated for a grid of λ_1, which is chosen such that the number of edges is between 1% and 10% of the total number of possible edges. This range intends to capture only sparse networks. The averaged number of selected edges is plotted against the penalty parameter λ_1 in Figure 6. For all five data sets Figure 6 shows that the number of selected edges in the ER+ samples is comparable (over the selected range of λ_1) to the ER- samples. Thus, when DNA copy number is not taken into account, the gene-gene interactions of the regulatory network do not differ between the two ER groups.

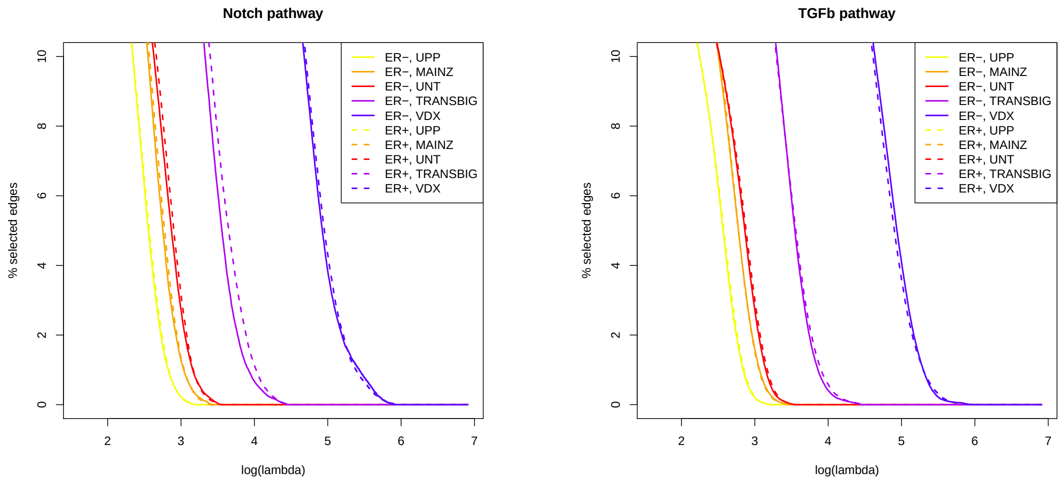

Figure 6: Number of edges present vs. penalty parameter, for ER- (solid lines) and ER+ (dashed lines) group for five 'transcriptome only' breast cancer studies. Left panel: Notch pathway; right panel: TGFβ pathway.

References

Barabási, A.-L. and Albert, R. (1999). Emergence of scaling in random networks. *Science*, **286**, 509–512.

Chin, K., DeVries, S., Fridlyand, J., Spellman, P. T., Roydasgupta, R., Kuo, W. L., Lapuk, A., Neve, R. M., Qian, Z., Ryder, T., Chen, F., Feiler, H., Tokuyasu, T., Kingsley, C., Dairkee, S., Meng, Z., Chew, K., Pinkel, D., Jain, A., Ljung, B. M., Esserman, L., Albertson, D. G., Waldman, F. M., and Gray, J. W. (2006). Genomic and transcriptional aberrations linked to breast cancer pathophysiologies. *Cancer Cell*, **10**, 529–541.

Csardi, G. and Nepusz, T. (2006). The igraph software package for complex network research. *InterJournal*, **Complex Systems**, 1695.

Harville, D. A. (2008). *Matrix Algebra From a Statistician's Perspective*. Springer, New York.

Horlings, H. M., Lai, C., Nuyten, D. S. A., Halfwerk, H., Kristel, P., van Beers, E., Joosse, S. A., Klijn, C., Nederlof, P. M., Reinders, M. J. T., Wessels, L. F. A., and Van de Vijver, M. J. (2010). Integration of DNA copy number alterations and prognostic gene expression signatures in breast cancer patients. *Clinical Cancer Research*, **16**(2), 651–663.

Klijn, C., Holstege, H., De Ridder, J., Liu, X., Reinders, M., Jonkers, J., and Wessels, L. (2008). Identification of cancer genes using a statistical framework for multiexperiment analysis of nondiscretized array CGH data. *Nucleic Acids Research*, **36**(2), e13.

Peng, J., Wang, P., Zhou, N., and Zhu, J. (2009). Partial correlation estimation by joint sparse regression models. *Journal of the American Statistical Association*, **104**(486), 735–746.

Van Wieringen, W. N., Unger, K., Leday, G. G. R., Krijgsman, O., De Menezes, R. X., Ylstra, B., and Van de Wiel, M. A. (2012). Matching of array CGH and gene expression microarray features for the purpose of integrative genomic analyses. *BMC Bioinformatics*, **13**(80).

Wang, Y., Klijn, G. M., Zhang, Y., Sieuwerts, A. M., Look, M. P., Yang, F., Talantov, D., Timmermans, M., Meijer-van Gelder, M. E., Yu, J., Jatkoe, T., Berns, E. M. J. J., Atkins, D., and Foekens, J. A. (2005). Gene-expression profiles to predict distant metastasis of lymph-node-negative primary breast cancer. *Lancet*, **365**(9460), 671–679.

Watts, D. J. and Strogatz, S. H. (1998). Collective dynamics of 'small world' networks. *Nature*, **393**, 440–442.

Zhang, Y., Martens, J. W. M., Yu, J. X., Jiang, J., Sieuwerts, A. M., Smid, M., Klijn, J. G. M., Wang, Y., and Foekens, J. A. (2009). Copy number alterations that predict metastatic capability of human breast cancer. *Cancer Research*, **69**(9), 3795–3801.
